# Beyond food and medicine, but necessary for life, too: other folk plant uses in several territories of Catalonia and the Balearic Islands

**DOI:** 10.1186/s13002-016-0097-8

**Published:** 2016-06-17

**Authors:** Airy Gras, Teresa Garnatje, M. Àngels Bonet, Esperança Carrió, Marina Mayans, Montse Parada, Montse Rigat, Joan Vallès

**Affiliations:** Laboratori de Botànica - Unitat associada CSIC, Facultat de Farmàcia, Universitat de Barcelona, Av. Joan XXIII s/n, 08028 Barcelona, Catalonia Spain; Institut Botànic de Barcelona (IBB–CSIC–ICUB), Passeig del Migdia s/n. Parc de Montjuïc, 08038 Barcelona, Catalonia Spain

**Keywords:** Balearic Islands, Catalonia, Ethnobotany, Non-food and non-medicinal plant uses

## Abstract

**Background:**

Ethnobotanical academic research, particularly in European industrialised countries, has been, and is, mostly focused on folk uses of food and medicinal plants. Nevertheless, other uses, as may well be supposed, account for a significant portion of these folk uses. In the Catalan linguistic domain, a considerable amount of ethnobotanical work has been produced, but to date almost nothing has been published on these other plant uses.

**Methods:**

We basically used the method of semistructured interviews to collect data on names, knowledge and use of plants in the above-mentioned fields from 759 informants in three Catalonian (Alt Empordà, Montseny and Ripollès) and two Balearic (Formentera and Mallorca) areas. We identified the plants quoted by the informants and prepared herbarium vouchers. We analysed and compared the results obtained.

**Results:**

Information has been collected on 401 genera, 552 species, 81 subspecies and four varieties, belonging to 122 families, totalling 4137 use reports for popular non-food and non-medicinal uses (classified in 14 modalities), and designated with 1303 folk Catalan names. The informant consensus factor is 0.87, accounting for a consistent and robust dataset.

**Conclusion:**

Contrarily to what could be thought a priori, and irrespective of the fact that some uses are declining or changing, non-medicinal and non-food folk plant uses strongly persist in the territories considered, are highly considered by their practitioners, and may even imply some economic revenues.

## Background

When coining the term ethnobotany, Harshberger [1] considered as basic points for the newly named science “elucidating the cultural position of tribes who used plants” (where, nowadays, ‘tribes’ is replaced by ‘human groups’, [2]), “clarifying the past distribution of plants”, “determining trade routes” and “suggesting new current production lines” for useful plants. For Portères and Barrau [3, 4], ethnobotany is a discipline located at the crossroad between natural and human sciences studying the behaviour of human societies with regard to plants. Other authors state that ethnobotanical research rescues and updates the history of plants in human societies through time and space [5].

Even if ethnobotany deals with all kinds of plant uses, at least in western European countries, ethnobotanical research focused on food and medicinal plants is by far the most dominant, what can be verified when surveying the articles that have appeared in relevant journals. Such a situation can be explained by two kinds of reasons. On the one hand, food and medicine are the two plant folk applications more directly linked to human health, so that they are maintained at a relatively high rate even in industrialised societies. Conversely, these countries have undergone a rather deep process of acculturation, in the sense of adopting so-called modern habits in detriment of traditional culture [6], which implied that rural communities abandoned a great deal of traditional practices linked to plants, because they are much less necessary (if necessary at all) nowadays than in the first half of 20^th^ century. On the other hand, the above-quoted food and medicinal properties are those which most likely can lead to the development of new commercial sources of welfare products (see e.g., [7]). Some non-food and non-medicinal popular uses are slightly better preserved than most of them, such as basketry [8] and cosmetics [9, 10], the latter being, in fact, very closely linked to medicinal ones. Some specific papers with ethnobotanical focus have been devoted to these other plant uses in European countries (e.g., those quoted for basketry and cosmetics, as well as [11–16]). Additionally, some compilation work, rather addressed to folk knowledge vulgarisation, contain information on these plant utilisations; as an example the first volume of the ongoing Spanish Inventory of Traditional Knowledge related to Biodiversity [17] provides information on all kinds of plant uses, including those dealt with in the present paper. In any case, even taking into account the above-quoted contributions, this approach still remains quite scarce.

The research on ethnobotany in the Catalan linguistic domain has been intense in the last 25 years. This research mostly comprises data on food and medicinal (including veterinarian) plant uses, as well as studies linked to agroecosystems (mostly homegardens) and to ethnoecological questions (cf. [10, 18], and references therein). Only very rarely other plant uses have been addressed [19, 20], although, throughout our research, we have maintained the conviction that a robust pool of knowledge on these uses still exists. Taking this into account, the aims of the present paper are: 1) to provide an overview on non-food and non-medicinal popular plant uses in five Catalan language territories, three in Catalonia and two in the Balearic Islands; 2) to evaluate to what extent these uses are persisting and how are they currently considered by their practitioners.

## Methods

### Study areas

**Fig. 1:**
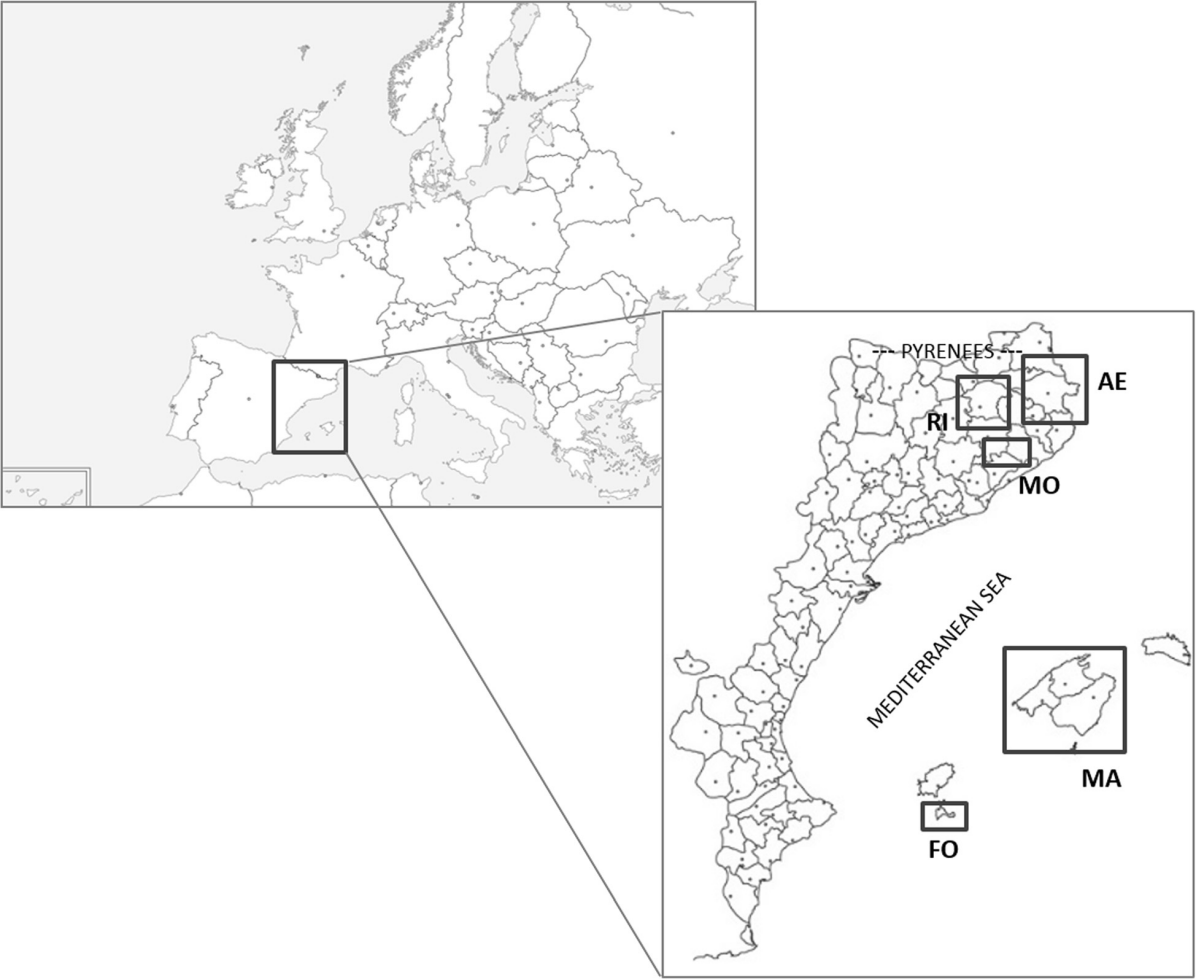
Studied areas in the contexts of Europe and the Catalan linguistic domain. AE: Alt Empordà; MO: Montseny; RI: Ripollès; MA: Mallorca; FO: Formentera

We performed interviews in five territories located in two large areas of the Catalan linguistic domain, three in Catalonia (Alt Empordà, Montseny, Ripollès) and two in the Balearic Islands (Formentera, Mallorca), with the aim of comparing a continental place and an insular one (Fig. 1). The Catalonian areas comprise from plain to high mountains, and the Balearic ones involve two islands.

Alt Empordà (AE) is a district (*comarca* in the Catalan language) of 1358 km^2^ and 140,214 inhabitants [21] living in 68 municipalities. The climate is mainly coastal Mediterranean. The vegetation is distributed in an asymmetrical form in two biogeographical regions, Mediterranean, largely dominant, and Eurosiberian, in certain mountainous areas, reaching 1443 m a.s.l.

Ripollès (RI) is a district occupying an area of 956.6 km^2^ and having a population of 25,700 inhabitants [21] distributed in 19 municipalities, with a high percentage of the population inhabiting small villages and isolated houses. Located in the eastern Pyrenees, it has a high mountain climate with Mediterranean influence. The flora and vegetation are mostly Eurosiberian, with some Boreoalpine zones in mountain areas reaching 2909.8 m a.s.l. Montseny (MO) is a mountain massif with a maximum altitude of 1706 m a.s.l., with an area of 826 km^2^ and a population of 105,000 people [22]. The climate is basically Mediterranean (including mountain Mediterranean). The vegetation belongs to the Mediterranean and Eurosiberian regions. Formentera (FO) is the smallest of the four inhabited Balearic Islands, occupying 82 km^2^, and has 11,545 inhabitants [23] living in nine population centres, all belonging to one municipality. Its maximum altitude is 195 m. The climate is Mediterranean with an arid tendency. The vegetation landscape is basically coastal Mediterranean.

Mallorca (MA) is the biggest island in the Balearic archipelago, and the seventh largest in the Mediterranean sea, with the highest altitude at 1445 m. It has an extension of 3622.54 km^2^ and population of 858,313 inhabitants [23]. Its climate is typically Mediterranean. The vegetation belongs mostly to the Mediterranean and, to a small part, to the Eurosiberian biogeographic regions.

### Informants

Information was obtained from 769 informants, selected on a snowball basis [24] for general ethnobotanical prospections in each of the areas studied. Of them, 510 were from Catalonia and 259 from Balearic Islands, distributed as follows: 160 from RI, born between 1915 and 1988, with an average age of 71.6 years; 178 from AE, with a mean age of 69.44 years, ranging from 23 to 95; 172 from MO, with a mean age of 66, ranging from 31 to 96; 235 from MA, with an average of 76 years, born between 1906 and 1981; and 24 from FO, born between 1920 and 1943. Concerning gender, 445 interviewees were women (57.87 %) and 324 men (42.13 %).

### Field work methods

Data were collected with the same method, but in different years in each territory: 1993–2000 (MO), 1995–2007 (AE), 2004–2012 (RI), 2009–2013 (MA) and 2011–2013 (FO). Ethnobotanical interviews were performed with a total of 769 informants (see Results and discussion section for details on distribution, gender and age). Taking into account the Code of Ethics of the International Society of Ethnobiology [25], we asked the interviewees’ informed consent to participate in the survey, to register the interviews, to take pictures and to use their images and information. The method used was basically the semistructured interview [26], with special care taken in not asking direct questions, which could coerce, condition or influence the informant’s answer. In several occasions group interviews were carried out, separating the answers of the different interviewees. Whenever possible, we collected the plants in question together with the informants or we observed with them those they had preserved at home or their preparations or the objects elaborated with them. The interviews, developed in Catalan, the common language of both interviewers and interviewees, were registered, also with the informants’ agreement (which was also solicited for taking pictures).

Plants were identified at specific or infraspecific levels with the aid of the *Flora manual dels Països Catalans* [27], and for family assignation we followed the Angiosperm Phylogeny Group ([28]; http://www.mobot.org/MOBOT/research/APweb/), as currently reflected in The plant list (http://www.theplantlist.org), also devoted to gymnosperms and ferns. Vouchers of every taxon with associated information have been prepared, collected following the legislation and avoiding protected areas, and deposited in the herbaria BCN, of the Centre de Documentació de Biodiversitat Vegetal, Universitat de Barcelona, and BC, of the Institut Botànic de Barcelona.

### Databasing

Once in the laboratory, the interviews were transcribed and introduced into our research team’s database (www.etnobotanica.cat, not open to public access, but punctually consultable on request to the corresponding or to the last authors). This database contains all ethnobotanical data (on medicinal, food and other uses) from our group and allows us to manage the results, to perform some calculations and to establish comparisons. Taking Cook’s Economic Botany data standard as a departing point [29], a huge effort to establish and nuance use categories (as well as parts of plants and kinds of preparation and use, the latter not relevant here, but in medicinal and food uses) has been performed, and it is reflected in Table 1.

**Table 1:** Non-food and non-medicinal folk plant uses in the territories of Alt Empordà, Montseny and Ripollès (Catalonia), and Formentera and Mallorca (Balearic Islands)

Taxon	Catalan vernacular names (a few Spanish, French and Arabic, indicated)	Area	Used part	Uses	Frequency
*Abelmoschus esculentus* (L.) Moench (Malvaceae) BC131944	Ocra	BI	Seed	Agrosilvopastoral management: agricultural/horticultural	1
*Abies alba* Mill. (Pinaceae) BCN3096	Avet	AE, MO	Aerial part. Stem. Whole plant	Magic and religious beliefs and practices Ornamental: bouquet elaboration Timber: boat manufacturing	3
*Abies nordmanniana* (Steven) Spach (Pinaceae) BC59249	Avet	MO	Whole plant	Ornamental: gardening	1
*Abies* ×*masjoanis* D.A. Soto, J.I.G. Viñas et E. Bujarrabal (Pinaceae) BCN35720	Avet de Masjoan, avet del Montseny	MO	Whole plant	Ornamental: gardening	3
*Acacia dealbata* Link (Mimosaceae) BCN29973	Mimosa	AE, MO	Aerial part. Leaf	Magic and religious beliefs and practices Ornamental: bouquet elaboration	3
*Acer campestre* L. (Aceraceae) BCN29613	Auró, auró blanc	AE, RI	Stem. Whole plant	Ludic Timber: furniture manufacturing	2
*Acer monspessulanum* L. (Aceraceae) BCN29613	Auró negre	AE	Stem	Ludic	1
*Acer opalus* Mill. subsp. *opalus* (Aceraceae) BCN29615	Blada, baladre	AE, MO	Stem. Whole plant	Agrosilvopastoral management: agricultural/horticultural Folk oral literature Ludic	4
*Achillea ageratum* L. (Asteraceae) BCN- E-188	Alè de bou	BI	Aerial part. Whole plant	Agrosilvopastoral management: tools for agricultural practices Magic and religious beliefs and practices Other informations: ecological information	3
*Achillea millefolium* L. (Asteraceae) BCN29616	Centfulles	AE	Aerial part	Ornamental: bouquet elaboration	1
*Achillea ptarmica* L. subsp. *pyrenaica* (Godr.) Heimerl (Asteraceae) BCN24701	Camamilla, camamilla de muntanya, camamilla de Rojà	RI	Aerial part	Cosmetic	1
*Adiantum capillus-veneris* L. (Pteridiaceae) BCN91889	Falzia	BI	Not reported. Whole plant	Cosmetic Other informations: excipient or adjuvant in medicinal or food preparations Other informations: use not remembered	3
*Aegilops geniculata* Roth (Poaceae) BCN 103555	Blat de perdiu	BI	Aerial part. Whole plant	Other informations: ecological information	2
*Aesculus hippocastanum* L. (Sapindaceae) BCN29618	Castanyer bord, castanyer d'Índies, castanya agra, castanya borda (fruit), castanya de castanyer d'Índies (fruit), morronyer	AE, BI, MO, RI	Fruit. Seed. Whole plant	Ludic Magic and religious beliefs and practices Ornamental: gardening	8
*Agapanthus* sp. (Amaryllidaceae)	Acapantus	BI	Whole plant	Ornamental: gardening	1
*Agave americana* L. (Agavaceae) BCN46860	Ágave, atzavara, donarda, figuerassa, figuerassa de marge, figuerassa grossa, pita, pitera, punta de pita (thorn), punyalera, xeremec (stem)	AE, BI, MO	Flower. Leaf, Not reported. Stem. Thorn	Agrosilvopastoral management: agricultural/horticultural Artisanal: cookware elaboration Artisanal: footwear elaboration Domestic: for help at home Textile: fibre or cloth elaboration Timber: construction materials production	18
*Agrimonia eupatoria* L. (Rosaceae) BCN24704	Agafallosa	RI	Fruit	Ludic	3
*Agrostemma githago* L. (Caryophyllaceae) BCN5737	Clavell de blat, niella	MO, RI	Aerial part. Whole plant	Folk oral literature Ornamental: bouquet elaboration	2
*Ailanthus altissima* (Mill.) Swingle (Simaroubaceae) BCN81396	Cornissa, irlando	MO	Whole plant	Ornamental: gardening	1
*Ajuga iva* (L.) Schreb. (Lamiaceae) BCN6437	Iva	BI	Whole plant	Folk oral literature	1
*Alkanna tinctoria* (L.) Tausch (Boraginaceae) BCN24706	Herba del peu de colom, peu de colom, pota de colom	RI	Root	Cosmetic	2
*Allium ampeloprasum* L. (Amaryllidaceae) BCN3993	All de bruixa, allassa, porrerell	AE, BI	Flower	Magic and religious beliefs and practices Other informations: ecological information	3
*Allium cepa* L. (Liliaceae) BCN28655	Ceba, ceba forastera, grell	AE, BI, MO	Bulb. Inflorescence	Agrosilvopastoral management: honey obtaining Domestic: for help at home Magic and religious beliefs and practices Ornamental: bouquet elaboration Other informations: ecological information Repellent	13
*Allium fistulosum* L. (Amaryllidaceae) BCN-E248	Cebollí	BI	Inflorescence	Ornamental: bouquet elaboration	1
*Allium porrum* L. (Amaryllidaceae) BCN28791	Porro	AE, BI	Aerial part. Whole plant	Agrosilvopastoral management: agricultural/horticultural Ornamental: bouquet elaboration	2
*Allium roseum* L. (Amaryllidaceae) BCN31259	All de bruixa, calabruix	AE	Whole plant	Other informations: ecological information	2
*Allium sativum* L. (Amaryllidaceae) BCN29832	All, all de Sant Pau, all pau	AE, BI, MO	Bulb. Inflorescence. Whole plant	Agrosilvopastoral management: agricultural/horticultural Folk oral literature Magic and religious beliefs and practices Ornamental : gardening Ornamental: bouquet elaboration Other informations: ecological information	18
*Allium triquetrum* L. (Amaryllidaceae) BCN95566	Vitrac	BI	Whole plant	Other informations: ecological information	1
*Alnus glutinosa* (L.) Gaertn. (Betulaceae) BCN29620	Vern	AE, MO, RI	Aerial part. Stem. Whole plant	Agrosilvopastoral management: for fishing Agrosilvopastoral management: for hunting Agrosilvopastoral management: tools for agricultural practices Artisanal: footwear elaboration Folk oral literature Magic and religious beliefs and practices Ornamental: bouquet elaboration	15
*Alocasia odora* (Lindl.) K.Koch (Araceae) BCN50215	Marquesa	AE, BI	Whole plant	Magic and religious beliefs and practices Ornamental: gardening Other informations: ecological information	3
*Aloe vera* (L.) Burm f. (Xanthorrhoeaceae) BCN27242	Aloe vera, àloe vera, cactus, figuerassa	AE, BI	Leaf juice. Not reported. Whole plant	Cosmetic Magic and religious beliefs and practices Other informations: ecological information	6
*Althaea rosea* (L.) Cav. (Malvaceae) BCN6470	Malva reial, vauma, vauma loca	BI, MO	Flower. Whole plant	Magic and religious beliefs and practices Ornamental: gardening	3
*Alyssum maritimum* (L.) Lam. (Brassicaceae) BCN29622	Alíssum, caps blancs, escopinya de Crist, flor de gat, herbamel	AE, BI, RI	Whole plant	Agrosilvopastoral management: tool for agricultural practices Magic and religious beliefs and practices Other informations: ecological information Other informations: use not remembered Repellent	5
*Amelanchier ovalis* Medik. (Rosaceae) BCN 16410	Cornier	RI	Aerial part	Ornamental: bouquet elaboration	2
*Ampelodesmos mauritanicus* (Poir.) T.Durand & Schinz (Poaceae) BCN95589	Càrritx, càrritx femella, càrritx mascle, carritxera	BI	Aerial part. Leaf. Stem. Whole plant	Agrosilvopastoral management: for pig slaughter Artisanal: basketry Other informations: ecological information Other informations: undesirable actions Textile: fibre or cloth elaboration	18
*Anacyclus clavatus* (Desf.) Pers. (Asteraceae) BCN31260	Bòlig	AE	Whole plant	Other informations: ecological information	1
*Anacyclus valentinus* L. (Asteraceae) BCN29625	Bòlig	AE	Whole plant	Other informations: ecological information	2
*Anagallis arvensis* L. subsp. *arvensis* (Primulaceae) BCN19974	Borró	AE	Whole plant	Other informations: ecological information	1
*Anagyris foetida* L. (Fabaceae) BCN16938	Garrover del dimoni	BI	Aerial part	Other informations: undesirable actions	1
*Anastatica hierochuntica* L. (Brassicaceae) BCN16949	Rosa de Jericó	BI	Whole plant	Other informations: use not remembered	1
*Anemone coronaria* L. (Ranunculaceae) BCN17399	Anèmones	BI	Aerial part	Ornamental: bouquet elaboration	1
*Anemone pulsatilla* L. (Ranunculaceae) BCN24711	Flor de Sant Pere	RI	Whole plant	Other informations: ecological information	2
*Anethum graveolens* L. (Apiaceae) BCN83632	Anet	BI	Whole plant	Ornamental: gardening	1
*Anthemis arvensis* L. (Asteraceae) BC840033	Bòlig, sistorna	RI	Aerial part	Ornamental: bouquet elaboration	1
*Anthemis cotula* L. (Asteraceae) BCN29835	Camamilla borda	AE	Whole plant	Other informations: ecological information	1
*Anthyllis cytisoides* L. (Fabaceae) BCN20700	Aubada, botja de cuques	BI	Aerial part	Agrosilvopastoral management: for pig slaughter Artisanal: broom elaboration Other informations: ecological information	5
*Antirrhinum majus* L. (Plantaginaceae) BCN46074	Badocs, boques de conill, conillets, gatets, gossets, gossos, gorges de llop, mamaconills, pets de llop	AE, MO, RI	Aerial part. Flower. Whole plant	Ludic Magic and religious beliefs and practices Ornamental: bouquet elaboration Ornamental: gardening	29
*Aphyllanthes monspelliensis* L. (Asparagaceae) BCN29627	Llonzella	AE	Aerial part	Ludic	1
*Apium graveolens* L. (Apiaceae) BCN46859	Àpit	BI	Leaf. Whole plant	Other informations: ecological information	2
*Aquilegia vulgaris* L. (Ranunculaceae) BCN27253	Campaneta	RI	Aerial part	Ornamental: bouquet elaboration	1
*Arachis hypogaea* L. (Fabaceae) BCN46858	Cacauet	AE, BI	Epicarp. Stem	Domestic: for help at home Fuel obtaining: charcoal	2
*Araucaria heterophylla* (Salisb.) Franco (Araucariaceae) BCN67780	Arbre de pisos	BI	Whole plant	Ornamental: gardening	1
*Araujia sericifera* Brot. (Apocynaceae) BCN299975	Miraguano	AE	Fruit	Textile: textile padding	3
*Arbutus unedo* L. (Ericaceae) BCN100976	Arboç, arboça (fruit), arbocera, cirera d'arboç (fruit), llipota (fruit), llipoter	BI, MO	Aerial part. Flower. Fruit. Not reported. Stem. Whole plant	Agrosilvopastoral management: forestry Agrosilvopastoral management: honey obtaining Fuel obtaining: charcoal Ornamental: bouquet elaboration Ornamental: gardening Other informations: ecological information Timber	10
*Arctium minus* (Hill) Bernh. (Asteraceae) BCN31262	Enganxadora, llapassa, llapissera, gafarró, poltre, repalassa	AE, MO, RI	Leaf. Inflorescence. Infructescence. Whole plant	Agrosilvopastoral management: tool for agricultural practices Ludic Other informations: ecological information	8
*Arisarum vulgare* O.Targ.Tozz. (Araceae) BCN95564	Frare llec, rapa, rapa de frare	BI	Inflorescence. Whole plant	Folk oral literature Ludic Other informations: ecological information	7
*Artemisia abrotanum* L. (Asteraceae) BCN31263	Absenta, abrótano macho (Spanish), artemisia, donzell, estragó, shiba (Arabic)	BI	Not reported	Cosmetic	1
*Artemisia absinthium* L. (Asteraceae) BCN29837	Absenta, abrótano macho (Spanish), artemisia, encens, donzell, estragó, shiba (Arabic)	AE, BI, RI	Aerial part. Whole plant	Domestic: air freshener Domestic: for help at home Repellent	3
*Artemisia arborescens* L. (Asteraceae) BCN29630	Artemisia, donzell	AE, BI	Aerial part. Whole plant	Agrosilvopastoral management: tool for agricultural practices Ornamental: bouquet elaboration Repellent	7
*Artemisia dracunculus* L. (Asteraceae) BCN13326	Estragó	BI	Whole plant	Other informations: ecological information	1
*Artemisia verlotiorum* Lamotte (Asteraceae) BCN29633	Artemisa, donzell	AE	Aerial part	Magic and religious beliefs and practices	1
*Artemisia vulgaris* L. (Asteraceae) BCN24719	Altimiris	RI	Aerial part	Agrosilvopastoral management: agricultural/horticultural	1
*Arum italicum* Mill. (Araceae) BCN32356	Xàrria	MO	Spathe	Ludic	1
*Arundo donax* L. (Poaceae) BCN29825	Canya, canya de torrent, canya mallorquina, canya verda, canyeta, canyís	AE, BI, MO, RI	Aerial part. Leaf. Root. Stem. Whole plant	Agrosilvopastoral management: for fishing Agrosilvopastoral management: for hunting Agrosilvopastoral management: for pig slaughter Agrosilvopastoral management: tools for agricultural practices Artisanal: basketry Artisanal: broom elaboration Artisanal: cane elaboration Artisanal: cookware elaboration Artisanal: musical instrument elaboration Artisanal: smoking pipe elaboration Domestic: for help at home Domestic: for sewing Fuel obtaining: firewood Ludic Magic and religious beliefs and practices Ornamental: gardening Other informations: ecological information Timber: construction materials production	104
*Asparagus acutifolius* L. (Asparagaceae) BCN29976	Espàrrec (turion), esparreguera	AE, BI, MO	Aerial part. Leaf. Stem. Whole plant	Agrosilvopastoral management: agricultural/horticultural Artisanal: broom elaboration Magic and religious beliefs and practices	4
*Asparagus albus* L. (Asparagaceae) BCN56215	Espàrrec (turion), esparreguera	BI	Aerial part	Ornamental: bouquet elaboration	1
*Asparagus horridus* L. (Asparagaceae) BCN103541	Espàrrec (turion), esparreguera, esparreguera vera	BI	Flower	Agrosilvopastoral management: honey obtaining	1
*Asparagus sprengeri* Regel (Asparagaceae) BCN57323	Esparreguera de jardí	BI	Whole plant	Ornamental: gardening	1
*Asphodelus aestivus* Brot. (Xanthorrhoeaceae) BCN52957	Albó, asfodel, aubó, caramutxa, coió de porrassa, moneiato de porrassera, porrassa, porrassa femella, porrassera, porassó	BI	Flower. Leaf. Stem. Tuber. Whole plant	Agrosilvopastoral management: for pig slaughter Agrosilvopastoral management: honey obtaining Agrosilvopastoral management: tools for agricultural practices Artisanal: footwear elaboration Domestic: for help at home Folk oral literature Fuel obtaining: firewood Magic and religious beliefs and practices Other information: harvesting and/or selling Other informations: ecological information Other information: use not remembered	33
*Aspidistra elatior* Blume (Asparagaceae) BCN104271	Cossiol de fulla, cossiol de saló, fulla de saló	BI	Whole plant	Ornamental: gardening	1
*Aspidosperma polyneuron* Müll.Arg. (Apocynaceae) BCN52088	Palorosa	AE	Stem	Artisanal: musical instrument elaboration	1
*Asplenium trichomanes* L. (Aspleniaceae) BCN24723	Auredelleta, costella de paret	RI	Aerial part	Cosmetics	1
*Aster novi-belgii* L. (Asteraceae) BCN98570	Setembrina	BI	Whole plant	Ornamental: gardening	2
*Aster pilosus* Willd. (Asteraceae) BCN47723	Octubre	AE	Aerial part	Ornamental: gardening	1
*Astragalus balearicus* Chater (Fabaceae) BCN95554	Coixinet de monja	BI	Whole plant	Other informations: ecological information	1
*Avena barbata* Pott ex Link (Poaceae) BCN49867	Cugula, cugula borda, civada borda	AE, BI, MO	Aerial part. Fruit. Inflorescence. Stem	Artisanal: musical instrument elaboration Ludic	10
*Avena sativa* L. (Poaceae) BCN32185	Civada	BI, MO	Fruit. Whole plant	Agrosilvopastoral management: agricultural/horticultural Magic and religious beliefs and practices Ornamental: bouquet elaboration Other informations: ecological information	9
*Avena sterilis* L. (Poaceae) BCN52787	Cugula, cugula borda, cugula vertadera	BI, MO	Aerial part. Inflorescence. Stem. Whole plant	Agrosilvopastoral management: agricultural/horticultural Artisanal: musical instrument elaboration Ludic	4
*Ballota hirsuta* Benth. (Lamiaceae) BCN72714	Sacramentària	BI	Calyx	Domestic: for help at home	1
*Begonia* sp. (Begoniaceae)	Begònia	BI	Whole plant	Ornamental: gardening	5
*Bellis annua* L. (Asteraceae) BCN61139	Confitera	BI	Whole plant	Other informations: ecological information	1
*Bellis perennis* L. (Asteraceae) BCN31264	Margarida, margarida borda, margaridoia, margarita	AE, MO, RI	Aerial part. Inflorescence. Whole plant	Magic and religious beliefs and practices Ornamental: bouquet elaboration Other informations: ecological information	7
*Berberis vulgaris* L. (Berberidaceae) BCN27274	Berbèlia	RI	Fruit	Ludic	1
*Beta vulgaris* L. subsp. *maritima* (L.) Arcang. (Amaranthaceae) BCN103432	Bledera salvatge	BI	Aerial part	Ornamental: bouquet elaboration	1
*Betula pendula* Roth (Betulaceae) BCN29647	Beç, bedoll	AE, RI	Aerial part. Leaf. Stem	Artisanal: broom elaboration Artisanal: cookware elaboration Magic and religious beliefs and practices	3
*Bidens tripartita* L. (Asteraceae) BCN50219	Pixallits	AE	Aerial part	Ornamental: bouquet elaboration	1
*Bignonia* sp. (Bignoniaceae)	Coral	BI	Whole plant	Ornamental: gardening	1
*Borago officinalis* L. (Boraginaceae) BCN68582	Borraja (Spanish), borratxa	BI, MO	Aerial part. Flower. Whole plant	Folk oral literature Ludic Ornamental: bouquet elaboration	3
*Bougainvillea* sp. (Nyctaginaceae)	Boquemvíl·lia	BI	Flower	Magic and religious beliefs and practices	1
*Brachypodium retusum* (Pers.) P.Beauv. (Poaceae) BCN31265	Llistó	AE	Stem. Whole plant	Agrosilvopastoral management: tools for agricultural practices Fuel obtaining: firewood Other informations: ecological information	4
*Brassica napus* L. (Brassicaceae) BCN24727	Nap	BI, RI	Bulb. Root	Magic and religious beliefs and practices	2
*Brassica oleracea* L. subsp. *oleracea* (Brassicaceae) BCN32181	Col	BI	Leaf	Folk oral literature Other informations: use not remembered	2
*Briza maxima* L. (Poaceae) BCN29648	Arrecades de la Mare de Déu, belluguets, herbe tremblante French, herba tremoluda	AE	Aerial part	Ornamental: bouquet elaboration	4
*Briza media* L. (Poaceae) BCN29650	Arrecadetes de la Mare de Déu, bellugadís, belluguets, bellugues, llagrimetes de la Mare de Déu, puces, sempreviva	AE, MO, RI	Aerial part	Ornamental: bouquet elaboration	9
*Bromus hordeaceus* L. (Poaceae) BCN52583	Cua de mula	MO	Aerial part	Ornamental: bouquet elaboration	1
*Buxus balearica* Lam. (Buxaceae) BCN21410	Boix	BI	Flower. Leaf. Stem. Whole plant	Artisanal: cane elaboration Artisanal: cookware elaboration Ornamental: gardening Other informations: harvesting and/or selling Other informations: ecological information Timber	11
*Buxus sempervirens* L. (Buxaceae) BCN29843	Boix, boix de jardí, boj (Spanish), olleta	AE, BI, MO, RI	Aerial part. Cortical parenchyma. Flower. Fruit. Leaf. Stem. Whole plant.	Agrosilvopastoral management: for pig slaughter Agrosilvopastoral management: tools for agricultural practices Artisanal: broom elaboration Artisanal: cane elaboration Artisanal: cookware elaboration Artisanal: musical instrument elaboration Artisanal: smoking pipe elaboration Domestic: for help at home Fuel obtaining: charcoal Ludic Magic and religious beliefs and practices Ornamental: bouquet elaboration Ornamental: gardening Other informations: use not remembered	127
*Calendula arvensis* M.Bieb. (Asteraceae) BCN29637	Llevamà, goig bord, jaumet	AE, BI	Aerial part. Not reported.Whole plant	Agrosilvopastoral management: tools for agricultural practices Cosmetic Folk oral literature Other informations: use not remembered	7
*Calendula officinalis* L. (Asteraceae) BCN29977	Boixac, bojac, calèndula, galdiró, llevamà, llevamà de jardí, jaumet	AE, BI, MO, RI	Aerial part. Inlforescence. Not reported. Whole plant	Cosmetic Magic and religious beliefs and practices Ornamental: bouquet elaboration Ornamental: gardening	14
*Calicotome spinosa* (L.) Link (Fabaceae) BCN29638	Argelac, argelaga	AE, BI	Aerial part. Stem. Whole plant	Agrosilvopastoral management: agricultural/horticultural Agrosilvopastoral management: for pig slaughter Ornamental: gardening	7
*Callistephus chinensis* (L.) Ness (Asteraceae) BCN-E249	Sol coronat	BI	Whole plant	Ornamental: gardening	1
*Calluna vulgaris* (L.) Hull (Ericaceae) BCN29639	Brossa, bruguerola, xerpó	AE, MO, RI	Aerial part. Flower	Agrosilvopastoral management: honey obtaining Ornamental: bouquet elaboration	13
*Camellia sinensis* (L.) Kuntze (Theaceae) BCN50762	Te	BI	Not reported	Other informations: ecological information	1
*Campanula patula* L. (Campanulaceae) BCN29640	Campaneta	AE	Aerial part	Ornamental: bouquet elaboration	1
*Campanula persicifolia* L. (Campanulaceae) BCN29641	Campaneta	AE	Aerial part	Ornamental: bouquet elaboration	1
*Cannabis sativa* L. (Cannabaceae) BCN24735	Cànem, cànyem, cànyom, gansalla (elaborated product), maria, marihuana	AE, BI, MO, RI	Aerial part. Inflorescence. Stem. Whole plant	Agrosilvopastoral management: for fishing Agrosilvopastoral management: tools for agricultural practices Artisanal: basketry Artisanal: footwear elaboration Other information: ecological information Smoking Textile: fibre or cloth elaboration Timber: boat manufacturing	17
*Capparis spinosa* L. subsp. *spinosa* (Capparaceae) BCN97808	Tàpera (flower bud), taperera	BI	Floral bud. Whole plant.	Folk oral literature Other informations: ecological information	5
*Capsella bursa-pastoris* (L.) Medik. (Brassicaceae) BCN46079	Pare i fill	BI	Not reported	Other informations	1
*Capsicum annuum* L. (Solanaceae) BCN24737	Pebre covent, pebre covent (fruit), pebre, pebrera, nyora, pebre vermell, pebrer, pebre de cirereta, pebresser, cirereta picant (fruit), pebre de banyeta (fruit), pebre (fruit)	BI	Fruit. Whole plant	Agrosilvopastoral management: agricultural/horticultural Magic and religious beliefs and practices Ornamental: bouquet elaboration Other informations: ecological information Repellent	14
*Capsicum* sp. (Solanaceae)	Pebre bord	BI	Seed	Agrosilvopastoral management: for pig slaughter	1
*Carlina acanthifolia* All. (Asteraceae) BCN24738	Carlina, carolina	RI	Aerial part. Whole plant	Agrosilvopastoral management: agricultural/horticultural Magic and religious beliefs and practices Ornamental Ornamental: bouquet elaboration Other informations: harvesting and/or selling Other informations: ecological information	14
*Carlina acanthifolia* All. subsp. *cynara* (Pourr. ex DC.) Rouy (Asteraceae) BCN46095	Carlina, carlina angèlica, carlinassa, carolina	AE, MO	Aerial part. Whole plant	Magic and religious beliefs and practices Other informations: ecological information	11
*Carlina acaulis* L. (Asteraceae) BCN32946	Carlina	AE	Whole plant	Other informations: ecological information	2
*Castanea sativa* Mill. (Fagaceae) BCN29844	Castanya (fruit), castanyer	AE, BI, MO, RI	Leaf. Inflorescence. Stem	Agrosilvopastoral management: for fishing Agrosilvopastoral management: tools for agricultural practices Artisanal: basketry Artisanal: cane elaboration Domestic: for help at home Ludic Ornamental: gardening Other informations: use not remembered Timber: boat manufacturing Timber: furniture manufacturing Timber: construction materials production	24
*Catananche caerulea* L. (Asteraceae) BCN29645	Cigales, pell de serp, pinya de plata	AE, RI	Aerial part. Inflorescence	Ludic Ornamental: bouquet elaboration	5
*Cedrus atlantica* (Endl.) Manetti ex Carrière (Pinaceae) BCN35722	Cedre	MO	Whole plant	Ornamental: gardening	1
*Cedrus libani* A.Rich. (Pinaceae) BCN29978	Cedre	AE	Stem	Timber: furniture manufacturing	1
*Celtis australis* L. (Cannabaceae) BCN29845	Lledoner, lledroner, lleroner, lledó (fruit), lledró (fruit), lleró (fruit)	AE, BI, MO	Fruit. Seed. Stem. Whole plant	Tools for agricultural practices Artisanal: broom elaboration Artisanal: cane elaboration Artisanal: musical instrument elaboration Ludic Magic and religious beliefs and practices Ornamental: gardening Other informations: ecological information Other informations: use not remembered Timber Timber: boat manufacturing	65
*Centaurea aspera* L. (Asteraceae) BCN42691	Bracera, herba de bracera	BI	Aerial part. Whole plant	Agrosilvopastoral management: tools for agricultural practices Other informations: ecological information	4
*Centaurea cyanus* L. (Asteraceae) BCN29651	Blauet, blau, caps blaus, clavell de blat, clavell de Sant Isidre, llums	AE, MO, RI	Aerial part. Inflorescence	Magic and religious beliefs and practices Ornamental: bouquet elaboration	4
*Centaurea pectinata* L. subsp. *pectinata* (Asteraceae) BCN43498	Barbassa, travalera, travarada	MO	Aerial part	Ludic	1
*Centranthus ruber* (L.) DC. subsp. *ruber* (Caprifoliaceae) BCN31267	Rosa d’Espanya	AE	Aerial part	Ornamental: bouquet elaboration	1
*Ceratonia siliqua* L. (Fabaceae) BCN32177	Garrofer, garrover, garrofa (fruit)	AE, BI, MO	Flower. Fruit. Seed. Whole plant	Agrosilvopastoral management: honey obtaining Artisanal: footwear elaboration Cosmetic Folk oral literature Fuel obtaining: firewood Other informations: harvesting and/or selling Other informations: ecological information Other informations: use not remembered Textile: dyer Timber: furniture manufacturing	21
*Cercis siliquastrum* L. (Fabaceae) BCN51020	Árbol de la pasión (Spanish), árbol del amor (Spanish)	BI	Whole plant	Ornamental: gardening	1
*Ceterach officinarum* Willd. (Aspleniaceae) BCN108285	Auradella	MO	Frond	Ornamental: bouquet elaboration	1
*Chamaerops humilis* L. (Arecaceae) BCN23832	Bri (leaflet), clin, garballó, garbeó, llatra (elaborated product), margalló, palma, palmito (fruit) (Spanish), pauma, paumera (group of palms), paumissó (fruit), pomera, pomissó (fruit), punissó (fruit)	AE, BI	Leaf. Leaflet. Not reported. Stem. Whole plant	Artisanal: basketry Artisanal: broom elaboration Domestic: for help at home Folk oral literature Magic and religious beliefs and practices Ornamental: bouquet elaboration Ornamental: gardening Other informations: ecological information Textile: fibre or cloth elaboration Textile: textile padding	49
*Chenopodium ambrosioides* L. (Amaranthaceae) BCN103590	Te mallorquí	BI	Whole plant	Other informations: appreciations on plant or product properties	1
*Chlorophytum comosum* (Thunb.) Jacques (Asparagaceae) BC617113	Cossiol de cintes	BI	Whole plant	Ornamental: gardening	1
*Chrysanthemum coronarium* L. (Asteraceae) BCN95559	Bòlig, sardonaia	BI	Inflorescence	Ludic	4
*Chrysanthemum frutescens* L. (Asteraceae) BCN93699	Margalida	BI	Whole plant	Ornamental: gardening	2
*Chrysanthemum segetum* L. (Asteraceae) BCN31269	Goit	AE	Whole plant	Other informations: ecological information	3
*Chrysanthemum sinense* Sabine (Asteraceae) BCN129085	Estranys	BI	Whole plant	Magic and religious beliefs and practices Repellent	2
*Cicer arietinum* L. (Fabaceae) BCN29659	Ciuró (seed)	BI	Whole plant	Agrosilvopastoral management: agricultural/horticultural	3
*Cichorium endivia* L. (Asteraceae) BCN46854	Endívia	BI	Leaf	Other informations: ecological information	1
*Cichorium intybus* L. (Asteraceae) BCN29660	Cama-rotja, cama-roja, masteguera	AE, BI	Leaf. Whole plant	Agrosilvopastoral management: agricultural/horticultural Folk oral literature Other informations: ecological information	13
*Cinnamomum camphora* (L.) J.Presl (Lauraceae) BCN50766	Càmfora (elaborated product)	AE, BI	Bark. Not reported	Magic and religious beliefs and practices Other informations: purchased in commerce Repellent	4
*Cirsium arvense* (L.) Scop. (Asteraceae) BCN29853	Calcida	RI, AE	Whole plant	Other informations: ecological information	3
*Cirsium vulgare* (Savi) Ten. (Asteraceae) BCN E-63	Card	MO	Inflorescence	Magic and religious beliefs and practices	1
*Cistus albidus* L. (Cistaceae) BCN36672	Estepa, estepa blanca, estepa d’escurar, estepa rosa	AE, BI, MO	Aerial part. Flower. Not reported. Stem. Whole plant	Agrosilvopastoral management: for pig slaughter Domestic: for help at home Fuel obtaining: firewood Ornamental: bouquet elaboration Other informations: ecological information	24
*Cistus monspeliensis* L. (Cistaceae) BCN36740	Estepa blanca, estepa borda, estepa de fulla llarga, estepa negra, mòdega	AE, MO	Flower. Resin. Stem. Whole plant.	Agrosilvopastoral management: tools for agricultural practices Artisanal: toy elaboration Domestic: for help at home Other informations: ecological information	6
*Cistus salviifolius* L. (Cistaceae) BCN36767	Estepa, estepa negra, estepa bona, estepa borrera, esteperola,	AE, BI, MO	Flower. Leaf. Stem. Whole plant	Agrosilvopastoral management: agricultural/horticultural Domestic: for help at home Other informations: ecological information Smoking	5
*Citrullus lanatus* (Thunb.) Matsum. & Nakai (Cucurbitaceae) BCN29662	Síndria (fruit), sindriera	BI	Fruit	Magic and religious beliefs and practices Folk oral literature	2
*Citrus* ×*aurantium* L. (Rutaceae) BCN46080	Taronja agra (fruit)	AE	Fruit	Agrosilvopastoral management: for pig slaughter	8
*Citrus grandis* (L.) Osbeck (Rutaceae) BCN52090	Pomelo (fruit)	AE	Whole plant	Other informations: ecological information	1
*Citrus limon* (L.) Osbeck (Rutaceae) BCN46853	Bergamota (fruit), llimona (fruit), llimoner, llimonera, mandarina (fruit), taronger bord, taronger clementí, taronger mandarí, taronja borda (fruit), taronja de la seca (fruit), taronja de pelar grillons (fruit), taronja de navel (fruit), pomelo (fruit)	AE, BI	Epicarp. Fruit. Fruit juice. Whole plant	Agrosilvopastoral management: for pig slaughter Cosmetic Domestic: air freshener Domestic: for help at home Folk oral literature Other informations: ecological information Repellent	23
*Citrus paradisi* Macfad. (Rutaceae) BCN-E250	Pomelo (fruit)	BI	Fruit	Other informations: use not remembered	1
*Citrus sinensis* (L.) Osbeck (Rutaceae) BCN24752	Taronger, taronja (fruit)	BI	Flower. Fruit. Leaf. Stem	Agrosilvopastoral management: for pig slaughter Artisanal: cane elaboration Artisanal: cookware elaboration Folk oral literature Ludic Magic and religious beliefs and practices Other informations: undesirable actions Timber	14
*Cladonia* sp. (Cladoniaceae)	Herba de la roca, herba roquera, musgo (Spanish)	BI	Not reported	Domestic: for help at home Magic and religious beliefs and practices	3
*Clematis cirrhosa* L. (Ranunculaceae) BCN22034	Vidauba, villauba	BI	Whole plant	Ornamental: bouquet elaboration Ornamental: gardening Other informations: use not remembered	3
*Clematis flammula* L. (Ranunculaceae) BCN29856	Didorta, joanillo, ridolta, ridorta, viadella, vidriella, vidauba	AE, BI, MO	Aerial part. Flower. Stem. Whole plan	Agrosilvopastoral management: tools for agricultural practices Domestic: for help at home Ornamental: bouquet elaboration Other informations: ecological information	8
*Clematis vitalba* L. (Ranunculaceae) BCN29857	Didorta, llidorta, ridolta, ridorta, vidiella	AE, MO, RI	Not reported. Stem. Whole plant	Agrosilvopastoral management: tools for agricultural practices Ludic Other informations: ecological information Smoking	18
*Cneorum tricoccon* L. (Rutaceae) BCN95567	Raspall	BI	Aerial part. Not reported	Artisanal: broom elaboration Folk oral literature	5
*Coffea arabica* L. (Rubiaceae) BCN-E-65	Cafè (seed)	MO	Seed	Magic and religious beliefs and practices	1
*Coix lacryma-jobi* L. (Poaceae) BC917450	Llàgrimes de Job, llàgrimes de viu	BI	Fruit	Folk oral literature Magic and religious beliefs and practices	2
*Colchicum autumnale* L. subsp. *autumnale* (Liliaceae) BCN45283	Cabeça	BI	Tuber	Ornamental: bouquet elaboration	1
*Colocasia* sp. (Araceae)	Colocàsia	BI	Whole plant	Folk oral literature Ornamental: gardening	2
*Colutea arborescens* L. subsp. *gallica* Browicz (Fabaceae) BCN56002	Pleps	MO	Stem	Ludic	1
*Conium maculatum* L. (Apiaceae) BCN32171	Cicuta	AE	Whole plant	Folk oral literature	1
*Consolida ajacis* (L.) Schur (Ranunculaceae) BCN60924	Espuela (Spanish)	MO	Flower. Whole plant	Magic and religious beliefs and practices Ornamental: gardening	3
*Convallaria majalis* L. (Liliaceae) BCN57362	Muguet	MO	Whole plant	Ornamental: gardening	2
*Convolvulus arvensis* L. (Convolvulaceae) BCN109798	Correjola, corretjola, corriola, corritjola	BI, MO	Flower. Whole plant	Agrosilvopastoral management: weeds Ludic Other informations: excipient or adjuvant in medicinal or food preparations	3
*Coriaria myrtifolia* L. (Coriariaceae) BCN24754	Rodó, roldor	RI	Leaf	Magic and religious beliefs and practices	1
*Coris monspeliensis* L. subsp. *monspeliensis* (Primulaceae) BCN110450	Farigola mascle, farigola de pastor, tomanyí	MO	Flower	Agrosilvopastoral management: honey obtaining	1
*Cornus sanguinea* L. (Cornaceae) BCN29979	Sanguinyol	AE	Sap	Magic and religious beliefs and practices	1
*Coronilla scorpioides* K.Koch (Fabaceae) BCN22206	Herba enamorada	BI	Whole plant	Folk oral literature	1
*Cortaderia selloana* (Schult. & Schult.f.) Asch. & Graebn. (Poaceae) BCN81361	Plomalls	MO	Aerial part	Ornamental: bouquet elaboration	1
*Corylus avellana* L. (Betulaceae) BCN29831	Avellana (fruit), avellaner, avellaner bord	AE, BI, MO	Leaf. Stem	Agrosilvopastoral management: tools for agricultural practices Artisanal: cane elaboration Magic and religious beliefs and practices Smoking Timber: construction materials production	12
*Crataegus azarolus* L. (Rosaceae) BCN14977	Atzerola (fruit), atzerolera	BI	Stem. Whole plant	Agrosilvopastoral management: tools for agricultural practices Timber	2
*Crataegus monogyna* Jacq. (Rosaceae) BCN103563	Cirerer de pastor, cireretes de pastor (fruit), espinal, espinaler, garganyer	BI	Whole plant	Agrosilvopastoral management: agricultural/horticultural Other informations: ecological information	5
*Crataegus monogyna* Jacq. subsp. *monogyna* (Rosaceae) BCN29858	Arç, arç blanc, arn, arn blanc	AE, MO, RI	Aerial part. Flower. Whole plant	Agrosilvopastoral management: tools for agricultural practices Folk oral literature Ornamental: bouquet elaboration	9
*Crithmum maritimum* L. (Apiaceae) BCN104272	Fonoll marí	BI	Aerial part	Folk oral literature Other informations: ecological information	2
*Crocus sativus* L. (Iridaceae) BCN32170	Safà	BI	Perigonium	Folk oral literature	1
*Cucumis melo* L. (Cucurbitaceae) BCN46851	Meló (fruit), moló (fruit), melonera, molonera,	BI	Epicarp. Fruit. Whole plant	Folk oral literature Ludic Magic and religious beliefs and practices Other informations: ecological information	5
*Cucumis sativus* L. (Cucurbitaceae) BCN46850	Cogombre, pepino (Spanish)	BI, MO	Fruit. Whole plant	Agrosilvopastoral management: agricultural/horticultural Cosmetic	2
*Cucurbita pepo* L. (Cucurbitaceae) BCN49858	Carabassera	BI	Flower. Fruit. Whole plant	Agrosilvopastoral management: agricultural/horticultural Folk oral literature Magic and religious beliefs and practices	4
*Cucurbita pepo* L. var. *oblonga* (Cucurbitaceae) BCN29859	Carabassó (fruit)	AE	Fruit	Ludic	2
*Cupressus macrocarpa* Hartw. (Cupressaceae) BCN35775	Ciprer	BI	Stem	Fuel obtaining: firewood	1
*Cupressus sempervirens* L. (Cupressaceae) BCN95563	Xifrer, xifrer baix, xifrer de fer tallavents, xiprer	AE, BI, RI	Fruictification. Whole plant	Agrosilvopastoral management: tools for agricultural practices Domestic: air freshener Magic and religious beliefs and practices Ornamental: gardening Other informations: ecological information	7
*Cuscuta epithymum* (L.) L. (Convolvulaceae) BCN112569	Tinya	RI	Whole plant	Folk oral literature	1
*Cydonia oblonga* Mill. (Rosaceae) BCN46849	Codony (fruit), codonyer	AE, BI, MO, RI	Fruit	Domestic: air freshener Other informations: ecological information	4
*Cynara cardunculus* L. (Asteraceae) BCN56003	Card gros, carxofera, card coler, card calapoter, carxofera borda, herba-col, presonera, presora	BI, MO	Aerial part. Whole plant	Folk oral literature Ornamental: bouquet elaboration	4
*Cynara scolymus* L. (Asteraceae) BCN24760	Carxofa (inflorescence), carxofera	BI	Leaf. Whole plant	Agrosilvopastoral management: tool for agricultural practices Other informations: ecological information	2
*Cynodon dactylon* (L.) Pers. (Poaceae) BCN24760	Gram, grama, xerpolla	BI, MO, RI	Aerial part. Whole plant	Agrosilvopastoral management: weeds Folk oral literature Magic and religious beliefs and practices Ornamental: gardening	5
*Cynoglossum creticum* Mill. (Boraginaceae) BCN97779	Llapassa, llapassera, llapissera	BI	Fruit. Leaf. Seed. Whole plant	Folk oral literature Other informations: ecological information Smoking	4
*Cyperus alternifolius* L. subsp. *flabelliformis* (Rottb.) M.R.Almeida (Cyperaceae) BCN 16451	Papiro (Spanish)	BI	Stem	Other informations: ecological information	1
*Cyperus eragrostis* Lam. (Cyperaceae) BCN61182	Castanyola, jonc, paraigüet, serrana	MO	Aerial part	Agrosilvopastoral management: tools for agricultural practices Ornamental: bouquet elaboration	3
*Cyperus rotundus* L. (Cyperaceae) BCN39997	Jonc	AE	Stem	Agrosilvopastoral management: tools for agricultural practices Artisanal: basketry	2
*Cystoseira amentacea* Bory var. *stricta* Mont. (Cystoseiraceae) BCN-Phyc-6232	Herba saupera	BI	Frond	Agrosilvopastoral management: for fishing	1
*Cystoseira crinita* Duby (Cystoseiraceae) BCN-Phyc-6233	Herba corretgera	BI	Frond	Agrosilvopastoral management: for fishing	1
*Cystoseira elegans* Sauv. (Cystoseiraceae) BCN-Phyc-6150	Herba borda	BI	Frond	Agrosilvopastoral management: for fishing	1
*Cytinus hypocistis* (L.) L. subsp. macranthus Wettst. (Rafflesiaceae) BCN2224	Magraneta, magraneta d’esteperol	BI	Not reported	Textile: dyer	4
*Dalbergia* sp. (Fabaceae) BCN52091	Palissandre	AE	Stem	Artisanal: musical instrument elaboration	1
*Dalhia* sp. (Asteraceae)	Dàlia, daliera	BI	Whole plant	Agrosilvopastoral management: tools for agricultural practices Ornamental: gardening	4
*Daphne gnidium* L. (Thymelaeaceae) BCN29687	Matapoll, tei, tintorell	AE, BI, MO	Bark. Root. Stem	Agrosilvopastoral management: for fishing Artisanal: basketry Artisanal: broom elaboration Domestic: for help at home Folk oral literature	9
*Datura stramonium* L. (Solanaceae) BCN29688	Castanyer bord, datura borda, estramoni, flor de mort, herba de capseta, herba talpera, herba taupera, matataups, trompetera	AE, BI, MO, RI	Aerial part. Leaf. Flower. Whole plant	Agrosilvopastoral management: agricultural/horticultural Magic and religious beliefs and practices Ornamental: gardening Other informations: ecological information Repellent	12
*Daucus carota* L. subsp. *carota* (Apiaceae) BCN48714	Botxa, caps blancs, carrota borda, fonollassa, julivert bord, julivert de galipau, moixos (inflorescence), pastanaga borda, rosella borda	AE, BI, MO	Aerial part. Inflorescence. Not reported	Agrosilvopastoral management: for pig slaughter Magic and religious beliefs and practices Ornamental: bouquet elaboration Other informations: ecological information	5
*Delphinium ajacis* L. (Ranunculaceae) BCN31271	Espuela (Spanish)	AE	Aerial part	Ornamental: bouquet elaboration	1
*Dianthus caryophyllus* L. (Caryophyllaceae) BCN31272	Clavell, clavell blanc, clavell d’hivernacle	AE, MO, RI	Aerial part. Flower	Magic and religious beliefs and practices Ornamental: bouquet elaboration	12
*Dianthus hyssopifolius* L. (Caryophyllaceae) BCN27299	Clavell	RI	Flower	Magic and religious beliefs and practices	1
*Dianthus seguieri* Vill. (Caryophyllaceae) BCN24763	Clavell de muntanya	RI	Flower	Magic and religious beliefs and practices	1
*Dianthus seguieri* Vill. subsp. *requienii* (Godr.) M.Bernal, Laínz & Muñoz Garm. (Caryophyllaceae) BCN22276	Clavell de bosc, clavellde pastor_	AE	Whole plant	Ornamental: bouquet elaboration	1
*Dianthus* sp. (Caryophyllaceae)	Clavell, clavell de Sant Jaume	BI, RI	Aerial part. Flower	Folk oral literature Ornamental: bouquet elaboration	3
*Dianthus pungens* L. subsp. *pungens* (Caryophyllaceae) BCN46087	Coixinet de monja	AE	Whole plant	Other informations: ecological information	1
*Diospyros ebenum* J.Koenig ex Retz. (Ebenaceae) BCN52092	Banús	AE	Stem	Artisanal: musical instrument elaboration	1
*Diospyros kaki* L.f. (Ebenaceae) BCN52627	Caqui (fruit), caquier, clequier	BI	Fruit. Stem	Agrosilvopastoral management: agricultural/horticultural Artisanal: musical instrument elaboration Other informations: excipient or adjuvant in medicinal or food preparations	3
*Diplotaxis erucoides* (L.) DC. (Brassicaceae) BCN29861	Cap blanc, ravenissa	AE	Whole plant	Other informations: ecological information	5
*Dipsacus fullonum* L. (Caprifoliaceae) BCN27266	Assotacristos, card, cardassa, cristos, escardot, escarpidora, escarpido, escarpín	BI, MO, RI	Aerial part. Flower. Inflorescence	Domestic: for help at home Ornamental: bouquet elaboration	10
*Dipsacus fullonum* L. subsp. *fullonum* (Caprifoliaceae) BCN29691	Cardó, cap de burro	AE	Aerial part	Ornamental: bouquet elaboration	3
*Dorycnium pentaphyllum* Scop. (Fabaceae) BCN103561	Botxa de cuques, socarrell, tijarell	BI	Aerial part. Leaf	Artisanal: broom elaboration Fuel obtaining: firewood	2
*Dorycnium pentaphyllum* Scop. subsp. *pentaphyllum* (Fabaceae) BCN115631	Botxa	MO	Flower	Agrosilvopastoral management: agricultural/horticultural	1
*Dryopteris filix-mas* (L.) Schott (Dryopteridaceae) BCN29692	Foguera, foguera de jardí	AE, MO	Frond. Whole plant	Ornamental: bouquet elaboration	3
*Ecballium elaterium* (L.) A.Rich. (Cucurbitaceae) BCF44970	Cobrombo silvestre, cogombre bord	BI, MO	Fruit. Whole plant	Ludic Other informations: ecological information	2
*Echinocactus grusonii* Hildm. (Cactaceae) BCN129080	Seient de sa sogra, grossini	BI	Whole plant	Ornamental: gardening	1
*Echinops ritro* L. subsp. *ritro* (Asteraceae) BCN31273	Espines blaves	AE	Aerial part	Ornamental: bouquet elaboration	5
*Emex spinosa* (L.) Campd. (Polygonaceae) BCN13757	Bledera borda, picatalons	BI	Fruit	Other informations: ecological information	1
*Epipremnum aureum* (Linden & André) G.S.Bunting (Araceae) BCN129081	Potos	BI	Whole plant	Ornamental: gardening	1
*Equisetum arvense* L. (Equisetaceae) BCN24767	Cua de cavall, escuraplata	RI	Aerial part	Domestic: for help at home	1
*Equisetum ramosissimum* Desf. (Equisetaceae) BCN29982	Coa de cavall, cua de cavall, cua de rata	AE, BI	Aerial part. Stem	Agrosilvopastoral management: tools for agricultural practices Other informations: ecological information	2
*Equisetum telmateia* Ehrh. (Equisetaceae) BCN118778	Coa de cavall, cova de cavall, cua de cavall, sangnua	BI, MO	Aerial part. Stem. Whole plant	Agrosilvopastoral management: tools for agricultural practices Cosmetic Ludic Magic and religious beliefs and practices Other informations: ecological information	5
*Erica arborea* L. (Ericaceae) BCN46081	Bruc, bruc bouer, bruc femella, bruc de fer escombres, bruc vertader, mascle	AE, MO, RI	Aerial part. Flower. Leaf. Stem	Agrosilvopastoral management: tools for agricultural practices Artisanal: broom elaboration Artisanal: cookware elaboration Artisanal: smoking pipe elaboration Fuel obtaining: charcoal Fuel obtaining: firewood Ludic Magic and religious beliefs and practices Domestic: for help at home Ornamental: bouquet elaboration Other informations: ecological information	58
*Erica multiflora* L. (Ericaceae) BCN29864	Bruc, bruc de bou, bruc de llei, bruc mascle, cepell, ciprell, ciprelló, petarrell, xiprell	AE, BI, MO	Aerial part. Flower. Stem. Whole plant	Agrosilvopastoral management: for pig slaughter Artisanal: broom elaboration Artisanal: smoking pipe elaboration Fuel obtaining: charcoal Fuel obtaining: firewood Magic and religious beliefs and practices Ornamental: bouquet elaboration Ornamental: gardening Other informations: ecological information Timber: construction materials production	33
*Erica scoparia* L. subsp. *scoparia* (Ericaceae) BCN46083	Bruc, bruc d’escombra, bruc de fer escombres, bruc de llei, bruc femella, bruga,	AE, MO	Aerial part. Stem	Artisanal: broom elaboration Artisanal: smoking pipe elaboration Ludic Ornamental: gardening	44
*Erigeron acer* L. (Asteraceae) BCN35105	_	MO	Inflorescence	Agrosilvopastoral management: agricultural/horticultural	1
*Eriobotrya japonica* (Thunb.) Lindl. (Rosaceae) BCN29695	Nisprero	BI	Leaf	Ornamental: bouquet elaboration Other informations: ecological information	2
*Erodium moschatum* (L.) L’Hér. (Geraniaceae) BCN95590	Rellotges	BI	Fruit	Ludic	3
*Eryngium bourgatii* Gouan (Apiaceae) BCN24881	Espinacal blau	RI	Aerial part	Ornamental: bouquet elaborations	1
*Eryngium campestre* L. (Apiaceae) BCN31274	Card, card girgoler, espinacal, espinacalç, espinacs, galabart, panical	AE, BI MO	Root. Whole plant	Agrosilvopastoral management: weeds Folk oral literature Magic and religious beliefs and practices Other informations: ecological information	6
*Eucalyptus globulus* Labill. (Myrtaceae) BCN29696	Calipto, calistro, calistrero, eucaliptus, eucalitus	AE, BI, MO	Aerial part. Flower. Leaf. Whole plant	Agrosilvopastoral management: forestry Agrosilvopastoral management: honey obtaining Domestic: air freshener Ornamental: bouquet elaboration	7
*Eupatorium cannabinum* L. subsp. *cannabinum* (Asteraceae) BCN35520	Altes i mires	MO	Whole plant	Ornamental: bouquet elaboration	1
*Euphorbia characias* L. subsp. *characias* (Euphorbiaceae) BCN29865	Lleteresa	AE	Stem. Whole plant	Repellent	2
*Euphorbia lathyris* L. (Euphorbiaceae) BCN29866	Cagamuix, herba talpera, herba taupera, herba taupinera	AE, RI	Latex. Leaf. Whole plant	Magic and religious beliefs and practices Repellent	6
*Euphorbia* sp. (Euphorbiaceae)	Eufòrbia, lletrada, lletrera	BI	Latex. Not reported	Agrosilvopastoral management: for hunting Artisanal: footwear elaboration Fuel obtaining: plant oil	3
*Fagus sylvatica* L. (Fagaceae) BCN46845	Faig	AE, BI, MO, RI	Stem. Whole plant	Agrosilvopastoral management: tools for agricultural practices Artisanal: musical instrument elaboration Fuel obtaining: charcoal Timber: boat manufacturing Timber: furniture manufacturing	15
*Feijoa sellowiana* O.Berg (Myrtaceae) BCN92988	Feijoa	BI	Whole plant	Ornamental: gardening	1
*Ferula communis* L. (Apiaceae) BCN26849	Canya fèl · lera, canyaferla	BI	Leaf. Stem	Ludic Other informations: ecological information	3
*Ficus carica* L. (Moraceae) BCN24887	Figa (infructescence), figa flor (infructescence), figó marinenc (infructescence) figuera, lletrada de figuera (latex), pàmpol de figuera	AE, BI	Bark. Infructescence. Stem. Whole plant	Agrosilvopastoral management: agricultural/horticultural Folk oral literature Ludic Magic and religious beliefs and practices Other informations: ecological information Other informations: excipient or adjuvant in medicinal or food preparationsSmoking Timber Timber: wheeled vehicles manufacturing	34
*Foeniculum vulgare* Mill. (Apiaceae) BCN95541	Fonoll	BI	Aerial part	Agrosilvopastoral management: for pig slaughter	1
*Foeniculum vulgare* Mill. subsp. *piperitum* (C.Presl) Ball (Apiaceae) BCN29867	Fonoll	AE	Leaf. Stem	Agrosilvopastoral management: for pig slaughter Domestic: for help at home	2
*Fraxinus angustifolia* Vahl (Oleaceae) BCN46844	Fleix, fleix femella, fleix mascle, freixa, freixe	AE, BI, MO	Stem. Whole plant	Agrosilvopastoral management: tools for agricultural practices Artisanal: broom elaboration Artisanal: cane elaboration Ludic Other informations: ecological information Timber: boat manufacturing Timber: wheeled vehicles manufacturing Timber: furniture manufacturing	21
*Fraxinus excelsior* L. (Oleaceae) BCN29698	Freixe	AE, MO, RI	Leaf. Stem. Whole plant	Artisanal: cane elaboration Artisanal: musical instrument elaboration Domestic: for help at home Magic and religious beliefs and practices Ornamental: gardening Timber: boat manufacturing Timber: wheeled vehicles manufacturing	11
*Fuchsia* sp. (Onagraceae)	Pendientes de la reina (Spanish)	BI	Whole plant	Ornamental: gardening	1
*Fumaria officinalis* L. (Papaveraceae) BCN72725	Fumaterra	BI	Whole plant	Other informations: harvesting and/or selling	1
*Gaillardia* sp. (Asteraceae)	Gallàrdia	BI	Whole plant	Ornamental: gardening	1
*Galanthus nivalis* L. (Amaryllidaceae) BCN27249	Flor de neu, herba de cap blanc, herba de neu, llàgrimes de Sant Josep	MO, RI	Aerial part	Ornamental: bouquet elaboration	5
*Galium aparine* L. (Rubiaceae) BCN97749	Rèvola	BI	Fruit	Other informations: ecological information	1
*Galium verum* L. (Rubiaceae) BCN39982	Herba de Sant Joan, santjoan	AE, RI	Aerial part. Not reported	Ornamental: bouquet elaboration Other informations: ecological information	2
*Genista tricuspidata* Desf. subsp. *sparsiflora* (Ball) Maire (Fabaceae) BCN 95565	Gatova	BI	Aerial part. Whole plant	Agrosilvopastoral management: for pig slaughter Folk oral literature	5
*Gentiana lutea* L. (Gentianaceae) BCN24893	Llenciana	RI	Aerial part. Root	Ornamental: bouquet elaboration Other informations: harvesting and/or selling	2
*Ginkgo biloba* L. (Ginkgoaceae) BCN62881	Ginkgo, ginkgo biloba	BI, MO	Whole plant	Ornamental: gardening Other informations: ecological information	2
*Gladiolus italicus* Mill. (Iridiaceae) BCN56421	Espadelles, espasa, espaseta, gladiol, xupes	BI, RI	Aerial part. Flower. Whole plant	Ornamental: bouquet elaboration Ornamental: gardening	5
*Gladiolus* sp. (Iridiaceae)	Gladiol	BI	Aerial part	Ornamental: bouquet elaboration	1
*Gleditsia triachanthos* L. (Fabaceae) BCN50767	Escàcia	AE	Stem	Domestic: for help at home	1
*Globularia alypum* L. (Globulariaceae) BCN29868	Cossiada, herba cossiada	BI	Leaf. Whole plant	Folk oral literature Other informations: ecological information	2
*Glycyrrhiza glabra* L. (Fabaceae) BCN47276	Regalíssia	AE	Root	Smoking	1
*Gomphocarpus fruticosus* (L.) W.T.Aiton (Asclepiadaceae) BCN29987	Tintorell	AE	Root	Agrosilvopastoral management: for fishing	1
*Gossypium* sp. (Malvaceae) BCN51688	Cotó	AE, BI	Fruit. Seed hair	Agrosilvopastoral management: for fishing Magic and religious beliefs and practices Other informations: ecological information	4
*Hedera helix* L. (Araliaceae) BCN29869	Heura	AE, BI, MO, RI	Aerial part. Flower. Leaf. Whole plant	Agrosilvopastoral management: honey obtaining Cosmetic Domestic: for help at home Magic and religious beliefs and practices Ornamental: gardening Other informations: use not remembered	22
*Helianthus tuberosus* L. (Asteraceae) BCN46069	Gira-sol de patata, miqueler, nyama, nyàmera, ramon, setembre, trumfa nyama	AE, BI MO, RI	Aerial part. Whole plant	Agrosilvopastoral management: tools for agricultural practices Ornamental: bouquet elaboration	6
*Helichrysum stoechas* (L.) Moench (Asteraceae) BCN29872	Éternelle (French), flor de Corpus, flor de Sant Joan, perpètua, sempreviva	AE, BI, MO	Aerial part. Inflorescence	Magic and religious beliefs and practices Ornamental: bouquet elaboration Repellent	38
*Helleborus foetidus* L. (Ranunculaceae) BCN32163	Margívol, marxívol	MO	Fruit. Leaf	Folk oral literature Ornamental: bouquet elaboration	9
*Helleborus viridis* L. (Ranunculaceae) BCN24899	Mengívol, marxívol	RI	Aerial part. Fruit	Magic and religious beliefs and practices	3
*Herniaria glabra* L. (Caryophyllaceae) BCN32161	Herba de cent en grana, santaengrana	MO	Whole plant	Folk oral literature	2
*Herniaria hirsuta* L. subsp. *cinerea* (DC.) Cout. (Caryophyllaceae) BCN45660	Herba de trencapreda, trencapedra	BI	Aerial part. Whole plant	Magic and religious beliefs and practices Other informations: ecological information	5
*Hibiscus syriacus* L. (Malvaceae) BCN47686	Altea	BI	Flower. Root. Whole plant	Ornamental: gardening Other informations: use not remembered	3
*Hippocrepis balearica* Jacq. (Fabaceae) BCN21809	Viola de penyal, violeta de penyal	BI	Whole plant	Agrosilvopastoral management: tools for agricultural practices Folk oral literature Ornamental: gardening	3
*Hirschfeldia incana* (L.) Lagr.-Foss. (Brassicaceae) BCN39984	Ravenissa	AE	Whole plant	Other informations: use not remembered	1
*Hordeum murinum* L. subsp. *leporinum* (Link) Arcang. (Poaceae) BCN79674	Cebada (Spanish), fletxa, malta, maragall, ordi	BI, MO	Inflorescence. Whole plant	Folk oral literature Ludic	8
*Hordeum vulgare* L. (Poaceae) BCN46843	Ordi	AE, BI, RI	Aerial part. Fruit. Stem. Whole plant	Agrosilvopastoral management: agricultural/horticultural Agrosilvopastoral management: honey obtaining Artisanal: footwear elaboration Cosmetic Magic and religious beliefs and practices Other informations: harvesting and/or selling	12
*Howea forsteriana* (F.Muell.) Becc. (Arecaceae) BCN129083	Quènsia	BI	Whole plant	Ornamental: gardening	1
*Hoya carnosa* (L.f.) R.Br. (Asclepiadaceae) BCN104268	Cerera	BI	Flower. Whole plant	Domestic: for help at home Ornamental: gardening	3
*Hydrangea macrophylla* (Thunb.) Ser. (Hydrangeaceae) BC800370	Hortènsia	MO	Flower	Magic and religious beliefs and practices	1
*Hydrangea* sp. (Hydrangeaceae)	Hortènsia	BI	Whole plant	Agrosilvopastoral management: tools for agricultural practices Ornamental: gardening	3
*Hyoscyamus albus* L. (Solanaceae) BCN 31277	Herba de capceta	BI	Leaf. Whole plant	Other informations: harvesting and/or selling Smoking	2
*Hypericum balearicum* L. (Clusiaceae) BCN95574	Espeta joana	BI	Aerial part. Whole plant	Other informations: ecological information Repellent	3
*Hypericum perforatum* L. (Clusiaceae) BCN29874	Herba de Sant Joan, herba dels set caps, hipèric, hipèricum, trescalam	AE, MO, RI	Aerial part. Not reported. Whole plant	Cosmetic Magic and religious beliefs and practices Ornamental: bouquet elaboration Other informations: harvesting and/or selling Other informations: purchased in commerce	8
*Ilex aquifolium* L. (Aquifoliaceae) BCN29876	Boix grèvol, flor de Nadal, grèvol	AE, MO, RI	Aerial part. Bark. Resin. Stem. Whole plant	Agrosilvopastoral management: tools for agricultural practices Agrosilvopastoral management: for hunting Artisanal: cane elaboration Magic and religious beliefs and practices Ornamental: bouquet elaboration Ornamental: gardening	25
*Inula viscosa* (L.) Aiton (Asteraceae) BCN31279	Herba de puces, matavinyes, olivarda	AE, BI	Aerial part. Inflorescence. Leaf. Not reported. Stem. Whole plant	Agrosilvopastoral management: for hunting Artisanal: broom elaboration Folk oral literature Magic and religious beliefs and practices Other informations: ecological information Other informations: use not remembered Repellent	15
*Ipomoea batatas* (L.) Lam. (Convolvulaceae) BCN50213	Monais, moneiato, moniato, moniajo	BI	Tuber. Whole plant	Agrosilvopastoral management: agricultural/horticultural Folk oral literature Ornamental: bouquet elaboration Other informations: ecological information	4
*Iris* ×*germanica* L. (Iridaceae) BCN31278	Lliri, lliri blau, lliri de marge, lliri de Sant Antoni, sords	AE, MO,RI	Aerial part. Flower. Whole plant	Magic and religious beliefs and practices Ornamental: bouquet elaboration Ornamental: gardening	8
*Iris pseudacorus* L. (Iridaceae) BCN31280	Lliri de rec	AE	Aerial part	Ornamental: bouquet elaboration	1
*Jacaranda* sp. (Bignoniaceae)	Xicaranda	BI	Whole plant	Ornamental: gardening	1
*Jasminum azoricum* L. (Oleaceae) BCN49864	Jasmí	AE	Whole plant	Repellent	1
*Jasminum officinale* L. (Oleaceae) BCN100423	Bejamí, gessamí, geramí, llessamí	BI, MO	Whole plant	Ornamental: gardening Other informations: ecological information	3
*Juglans regia* L. (Juglandaceae) BCN29877	Anoguer, anou (fruit), noguer, noguera, nou (fruit), nou verda (fruit), nogalina (elaborated product)	AE, BI, MO, RI	Endocarp. Fruit. Leaf. Stem. Whole plant	Agrosilvopastoral management: tools for agricultural practices Artisanal: cookware elaboration Cosmetic Domestic: for help at home Folk oral literature Magic and religious beliefs and practices Other informations: ecological information Textile: dyer Timber Timber: boat manufacturing Timber: furniture manufacturing	26
*Juncus effussus* L. (Juncaceae) BCN39991	Jonc, jonc marí	AE, MO	Aerial part. Stem	Agrosilvopastoral management: for fishing Artisanal: basketry Domestic: for help at home	9
*Juniperus communis* L. (Cupressaceae) BCN24910	Ginebre, ginebró, ginebró de jardí, oli de ginebre (elaborated product)	BI, MO, RI	Fructification. Stem. Whole plant	Agrosilvopastoral management: agricultural/horticultural Domestic: for help at home Folk oral literature Ornamental: gardening Other informations	13
*Juniperus communis* L. subsp. *communis* (Cupressaceae) BCN29878	Ginebre, ginebró	AE	Aerial part. Fruictification. Stem	Agrosilvopastoral management: tools for agricultural practices Magic and religious beliefs and practices Other informations: ecological information	3
*Juniperus oxycedrus* L. (Cupressaceae) BCN29879	Càdec	AE	Whole plant	Other informations: ecological information	1
*Juniperus oxycedrus* L. subsp. *oxycedrus* (Cupressaceae) BCN29879	Ginebre, ginebró	BI	Fruictification. Not reported. Root. Stem. Whole plant	Artisanal: musical instrument elaboration Artisanal: smoking pipe elaboration Domestic: for help at home Fuel obtaining: firewood Ornamental: gardening Other informations: ecological information	13
*Juniperus phoenicea* L. (Cupressaceae) BCN36078	Savina, savinó (cone), sivina	BI	Fruictification. Stem	Agrosilvopastoral management: tools for agricultural practices Artisanal: cane elaboration Ludic Timber	7
*Koelreuteria paniculata* Laxm. (Sapindaceae) BCN63272	Saboner	BI	Seed	Domestic: for help at home	1
*Lagenaria siceraria* (Molina) Standl. (Cucurbitaceae) BCN50212	Carabassa, catija	AE, BI	Fruit	Agrosilvopastoral management: tools for agricultural practices Artisanal: cookware elaboration Domestic: for help at home Ornamental: bouquet elaboration	5
*Lagurus ovatus* L. (Poaceae) BCN31281	_	AE	Aerial part	Ornamental: bouquet elaboration	2
*Lantana camara* L. (Verbenaceae) BCN 40081	Bandera espanyola	BI	Whole plant	Ornamental: gardening	2
*Lathyrus sativus* L. (Fabaceae) BCN 50775	Guixa (fruit)	BI	Whole plant	Agrosilvopastoral management: agricultural/horticultural	1
*Launaea cervicornis* (Boiss.) Font Quer & Rothm. (Asteraceae) BCN88711	Gatovell	BI	Aerial part	Agrosilvopastoral management: tools for agricultural practices	1
*Laurus nobilis* L. (Lauraceae) BCN29880	Llaurer, llor, llorer, llort	AE, BI, MO, RI	Aerial part. Leaf. Seed. Stem. Whole plant	Agrosilvopastoral management: tools for agricultural practices Artisanal: cane elaboration Cosmetic Domestic: air freshener Domestic: for help at home Folk oral literature Magic and religious beliefs and practices Other informations: ecological information Repellent Timber: construction materials production	49
*Lavandula angustifolia* Mill. (Lamiaceae) BCN29881	Espígol, lavanda	AE, BI	Aerial part. Flower. Whole plant	Domestic: air freshener Ornamental: bouquet elaboration Ornamental: gardening Other informations: ecological information	4
*Lavandula angustifolia* Mill. subsp. *angustifolia* (Lamiaceae) BCN29715	Barballó, bermelló, espígol, lavanda	RI	Aerial part	Domestic: air freshener	2
*Lavandula dentata* L. (Lamiaceae) BCN24913	Candelero, colònia, lavanda, gal·landa	AE, BI, MO	Aerial part. Flower. Whole plant	Agrosilvopastoral management: tools for agricultural practices Cosmetic Domestic: air freshener	9
*Lavandula latifolia* Medik. (Lamiaceae) BCN29882	Barballó, bermelló, espígol, lavanda	AE, BI, MO	Aerial part. Flower. Leaf. Whole plant	Agrosilvopastoral management: honey obtaining Agrosilvopastoral management: tools for agricultural practices Cosmetic Domestic: air freshener Ornamental: bouquet elaboration Ornamental: gardening Repellent Smoking	30
*Lavandula stoechas* L. (Lamiaceae) BCN126622	Cap d’ase, lavanda, tomanyí	BI, MO	Aerial part. Flower	Agrosilvopastoral management: tools for agricultural practices Cosmetic Smoking	7
*Lavandula stoechas* L. subsp. *stoechas* (Lamiaceae) BCN29883	Timó	AE	Aerial part. Flower	Domestic: air freshener Ornamental: bouquet elaboration Smoking	4
*Lavatera arborea* L. (Malvaceae) BCN95546	Malva	AE, BI	Aerial part. Flower	Agrosilvopastoral management: tools for agricultural practices Magic and religious beliefs and practices	2
*Lavatera cretica* L. (Malvaceae) BCN44944	Vauma	BI	Whole plant	Agrosilvopastoral management: weeds Folk oral literature Other informations: ecological information	4
*Lens culinaris* Medik. (Fabaceae) BCN 29990	Llentia	BI	Whole plant	Agrosilvopastoral management: agricultural/horticultural	1
*Leontodon taraxacoides* Hoppe & Hornsch. (Asteraceae) BCN48777	Angelets, pixallits	MO	Aerial part	Ludic	1
*Leontopodium alpinum* Guss. subsp. *alpinum* (Asteraceae) BCN34286	Herba de neu	AE	Aerial part	Ornamental: bouquet elaboration	1
*Leucanthemum vulgare* (Vaill.) Lam (Asteraceae) BCN29721	Margarida, margarida borda, margarida de camp, margarida de bosc	AE, RI	Aerial part	Ludic Ornamental: bouquet elaboration	4
*Leuzea conifera* (L.) DC. in Lam. & DC. (Asteraceae) BCN29884	Cigales, nyigo-nyigo, pinya de Sant Joan, polles	AE, MO	Aerial part	Ornamental: bouquet elaboration	4
*Ligustrum vulgare* L. (Oleaceae) BCN24915	Escanyacabres, olivereta	RI	Aerial part	Magic and religious beliefs and practices	1
*Lilium candidum* L. (Liliaceae) BCN46841	Azucena (Spanish), lliri, lliri blanc, lliri blanc de Sant Joan, lliri de Sant Antoni, lliri de Sant Joan	AE, BI, MO, RI	Aerial part. Flower. Whole plant	Magic and religious beliefs and practices Ornamental: bouquet elaboration Ornamental: gardening	10
*Lilium martagon* L. (Liliaceae) BCN24917	Consolta, macròlic, marcòlic vermell	MO, RI	Aerial part. Flower. Seedling	Ornamental: bouquet elaboration Ornamental: gardening	6
*Lilium pyrenaicum* Gouan (Liliaceae) BCN24918	Consolta, consolta groga, marcòlic groc	RI	Aerial part. Whole plant	Ornamental: bouquet elaboration Ornamental: gardening	3
*Limonium virgatum* (Willd.) Fourr. (Plumbaginaceae) BCN39989	Patarres	AE	Aerial part	Ornamental: bouquet elaboration	3
*Limonium vulgare* Mill. subsp. *serotinum* (Rchb.) Gams (Plumbaginaceae) BCN31282	Patarres	AE	Aerial part	Magic and religious beliefs and practices	1
*Linaria cymbalaria* (L.) Mill (Plantaginaceae) BCN29885	Picardia, setembrina	AE, BI, MO	Whole plant	Magic and religious beliefs and practices Ornamental: gardening	3
*Linum usitatissimum* L. (Linaceae) BCN47281	Lli, llinc, llim, llinosa, oli de llinosa (elaborated product)	AE, BI	Aerial part. Not reported. Seed. Seed oil. Whole plant	Domestic: paint elaboration Folk oral literature Textile: fibre or cloth elaboration	9
*Linum usitatissimum* L. subsp. *angustifolia* (Huds.) Thell. (Linaceae) BCN107485	Herba feridora	MO	Seed	Ornamental: bouquet elaboration	1
*Linum usitatissimum* L. subsp. *usitatissimum* (Linaceae) BCN32155	Bri (fibre), lli, llinosa	MO	Aerial part	Cosmetic	1
*Lippia triphylla* (L’Hér.) O.Kuntze (Verbenaceae) BCN29886	Herba lluïsa, marialluïsa	AE, BI	Aerial part. Leaf. Whole plant	Agrosilvopastoral management: agricultural/horticultural Domestic: air freshener Other informations: ecological information Other informations: excipient or adjuvant in medicinal or food preparations	8
*Lolium rigidum* Gaudin (Poaceae) BCN31283	Margai, margall	BI	Not reported	Folk oral literature	1
*Lonicera etrusca* Santi (Caprifoliaceae) BCN31285	Xuclamel	AE	Stem	Artisanal: smoking pipe elaboration	2
*Lonicera implexa* Aiton (Caprifoliaceae) BCN31286	Mistreres, lligabosc, madreselva (Spanish), mataselva, rotaboc, rotaboig, xuclamel	AE, BI, MO	Aerial part. Flower. Not reported. Stem. Whole plant	Agrosilvopastoral management: tools for agricultural practices Artisanal: smoking pipe elaboration Folk oral literature Ludic Ornamental: gardening Other informations: ecological information Other informations: use not remembered Repellent Smoking	19
*Lonicera peryclimenum* L. (Caprifoliaceae) BCN26736	Lligabosc, mareselva	MO	Flower	Magic and religious beliefs and practices Ornamental: bouquet elaboration	2
*Lophophora williamsii* (Lem. ex Salm-Dyck) J.M. Coult. (Cactaceae) BCN129082	Peyote (Spanish)	BI	Aerial part. Whole plant	Magic and religious beliefs and practices Ornamental: gardening Other informations: harvesting and/or selling	5
*Luffa cylindrica* (L.) M.Roem. (Cucurbitaceae) BCN-E-78	Carabassera d’esponges, fregall (fruit), fregall de carabassera (endocarp), fregaller	BI, MO	Endocarp. Fruit. Mesocarp + endocarp	Domestic: for help at home	4
*Lunaria annua* L. (Brassicaceae) BCN56009	Flor de plata	RI	Aerial part	Ornamental: bouquet elaboration	1
*Lunaria annua* L. subsp. *annua* (Brassicaceae) BCN31287	Flor de plata, medallons, moneda del Papa	AE, MO	Aerial part. Whole plant	Ornamental: bouquet elaboration	14
*Lupinus albus* L. (Fabaceae) BCN-E-79	Llobins, tramusset, tramús	MO	Seed. Whole plant	Agrosilvopastoral management: agricultural/horticultural Domestic: for help at home	3
*Lycopodium clavatum* L. (Lycopodiaceae) BCN31748	Musgo (Spanish)	BI	Aerial part	Agrosilvopastoral management: tools for agricultural practices	1
*Lygeum spartum* Loefl. ex L. (Poaceae) BCN 27831	Espart	BI	Not reported	Artisanal: basketry	1
*Magnolia grandiflora* L. (Magnoliaceae) BCN64396	Magnòlia	MO	Flower. Leaf. Whole plant	Magic and religious beliefs and practices Ornamental: gardening	3
*Malva sylvestris* L. (Malvaceae) BCN29889	Malva, malva de prat, vauma	AE, BI, MO, RI	Aerial part. Flower. Leaf. Whole plant	Folk oral literature Magic and religious beliefs and practices Other informations: ecological information	6
*Mammillaria* sp. (Cactaceae)	Mamillària	BI	Not reported. Whole plant	Ornamental: gardening Other informations: use not remembered	2
*Mandragora autumnalis* Bertol. (Solanaceae) BCN52434	Mandràgora	BI	Whole plant	Folk oral literature	1
*Mantisalca salmantica* (L.) Briq. & Cavill. (Asteraceae) BCN24925	Baleges, cabeçuda, granera	RI	Aerial part	Artisanal: broom elaboration Domestic: for help at home	4
*Marrubium vulgare* L. (Lamiaceae) BCN72729	Barrombí, malrobí	BI	Aerial part. Seed	Agrosilvopastoral management: honey obtaining Domestic: for help at home Magic and religious beliefs and practices Other informations: ecological information Repellent	5
*Matricaria chamomilla* L. (Asteraceae) BCN29890	Camamilla, camamilla de l’hort, camamilla dolça, mançanilla	AE, MO, RI	Aerial part. Inflorescence	Cosmetic Ornamental: bouquet elaboration	4
*Matthiola sinuata* (L.) R.Br. (Brassicaceae) BCN50218	Violer	AE	Aerial part	Ornamental: bouquet elaboration Ornamental: gardening	4
*Medicago sativa* L. (Fabaceae) BCN29891	Alfals, aufaus, userda	AE, BI, MO	Flower. Stem. Whole plant	Agrosilvopastoral management: agricultural/horticultural Domestic: for help at home Magic and religious beliefs and practices Other informations: ecological information Smoking	11
*Melia azedarach* L. (Meliaceae) BCN97016	Amèlia, sabonera	BI	Fruit	Magic and religious beliefs and practices	1
*Melilotus sulcatus* Desf. (Fabaceae) BCN27973	Trèvol de ramellet	BI	Flower	Other informations: ecological information	1
*Melissa officinalis* L. (Lamiaceae) BCN29731	Tarongí	BI	Aerial part. Leaf. Whole plant	Agrosilvopastoral management: tools for agricultural practices Domestic: air freshener Other informations: ecological information Repellent	6
*Melissa officinalis* L. subsp. *officinalis* (Lamiaceae) BCN29731	Tarongina	AE	Leaf	Cosmetic	1
*Mentha aquatica* L. (Lamiaceae) BCN103554	Menta d’aigua, menta de cavall	BI	Leaf	Other informations: excipient or adjuvant in medicinal or food preparations	1
*Mentha pulegium* L. (Lamiaceae) BCN29895	Poliol	AE, BI	Aerial part. Not reported	Ornamental: bouquet elaboration Other informations: use not remembered	2
*Mentha* sp. (Lamiaceae)	Herba-sana, hierba buena (Spanish), menta	BI, MO	Aerial part. Not reported. Whole plant	Folk oral literature Ornamental: bouquet elaboration Other informations: ecological information	5
*Mentha spicata* L. (Lamiaceae) BCN29995	Herba-sana, menta	AE, BI	Leaf. Whole plant	Domestic: for help at home Other informations: ecological information Repellent Smoking	8
*Mentha* × *piperita* L. (Lamiaceae) BCN29997	Menta piperita, pampalei, piperita, pipermint	BI	Leaf. Whole plant	Domestic: for help at home Other informations: ecological information	3
*Mespilus germanica* L. (Rosaceae) BCN50768	Nespla (fruit), nespler, nesplera	BI, MO	Fruit. Stem. Whole plant	Folk oral literature Other informations: ecological information Timber	5
*Mirabilis jalapa* L. (Nyctaginaceae) BCN126625	Buenas noches (Spanish), dama de nit, diegos de nit, noches (Spanish), Juan de noche (Spanish), sanjoans	MO, RI		Ornamental: gardening	3
*Morus alba* L. (Moraceae) BCN52588	Morer, morera	BI	Leaf. Stem	Other informations: excipient or adjuvant in medicinal or food preparations Other informations: appreciations on plant or product properties Timber: furniture manufacturing	3
*Muscari comosum* (L.) Mill. (Asparagaceae) BCN29738	Calabruix	AE	Whole plant	Other informations: ecological information	1
*Myrtus communis* L. (Myrtaceae) BCN35897	Multra, multrera, murta, murtera, murtó (fruit)	AE, BI	Aerial part. Flower. Leaf. Stem. Whole plant	Agrosilvopastoral management: tools for agricultural practices Agrosilvopastoral management: for fishing Artisanal: basketry Artisanal: tannery Cosmetic Folk oral literature Magic and religious beliefs and practices Ornamental: bouquet elaboration Other informations: ecological information Repellent	39
*Narcissus poeticus* L. (Amaryllidaceae) BCN47870	Jonquillo, lliri, lliri blanc, lliri de la Mare de Déu, narcís	MO	Whole plant	Ornamental: bouquet elaboration	7
*Narcissus pseudonarcissus* L. (Amaryllidaceae) BCN47849	Puigestela	RI	Aerial part	Ornamental: bouquet elaboration Ornamental: gardening	2
*Nepeta cataria* L. (Lamiaceae) BCN24935	Herba dels moixos, nepeta, nepta	BI, MO, RI	Aerial part. Whole plant	Agrosilvopastoral management: tools for agricultural practices Folk oral literature	4
*Nerium oleander* L. (Apocynaceae) BCN46840	Adelfa (Spanish), baladre	AE, BI, MO	Leaf. Stem. Whole plant	Agrosilvopastoral management: tools for agricultural practices Artisanal: musical instrument elaboration Magic and religious beliefs and practices Ornamental: gardening Repellent	13
*Nicotiana glauca* Graham (Solanaceae) BCN81462	Tabac fulla d’espasa	BI	Leaf	Smoking	1
*Nicotiana rustica* L. (Solanaceae) BCN46839	Tabac de pota, tabac pota	BI	Leaf. Whole plant	Other informations: ecological information Repellent Smoking	4
*Nicotiana tabacum* L. (Solanaceae) BCN48711	Caliquenya (elaborated product), tabac, tabac pota, tabaquera	AE, BI, MO	Leaf. Whole plant	Domestic: for help at home Other informations: purchased in commerce Repellent Smoking	17
*Ocimum basilicum* L. (Lamiaceae) BCN29897	Alfabaguera, alfàbrega, aufabeguera, albahaca (Spanish), enflàgam	AE, BI, RI	Aerial part. Leaf. Whole plant	Agrosilvopastoral management: tools for agricultural practices Cosmetic Domestic: air freshener Ludic Magic and religious beliefs and practices Ornamental: gardening Other informations: ecological information Repellent	52
*Odontites lutea* (L.) Clairv. (Orobanchaceae) BCN73750	Brancadella	MO	Flower	Agrosilvopastoral management: tools for agricultural practices	1
*Olea europaea* L. subsp. *europaea* (Oleaceae) BCN29898	Oli d’oliva (elaborated product), oliva (fruit), olivera	AE, BI, MO, RI	Aerial part. Fruit. Fruit juice. Not reported. Seed fruit. Stem. Whole plant	Artisanal: basketry Artisanal: cane elaboration Cosmetic Domestic: for help at home Folk oral literature Fuel obtaining: firewood Fuel obtaining: plant oil Ludic Magic and religious beliefs and practices Ornamental: bouquet elaboration Other informations: ecological information Other informations: excipient or adjuvant in medicinal or food preparations Timber Timber: boat manufacturing Timber: furniture manufacturing	82
*Olea europaea* L. subsp. *sylvestris* (Mill.). Lehr. (Oleaceae) BCN46838	Engís, olivera, olivera borda, ullastre	AE, BI	Aerial part. Leaf. Stem. Whole plant	Agrosilvopastoral management: agricultural/horticultural Agrosilvopastoral management: for fishing Agrosilvopastoral management: for hunting Artisanal: basketry Artisanal: cane elaboration Artisanal: musical instrument elaboration Domestic: for help at home Fuel obtaining: charcoal Fuel obtaining: firewood Ludic Magic and religious beliefs and practices Other informations: appreciations on plant or product properties Timber Timber: wheeled vehicles manufacturing Timber: furniture manufacturing	44
*Onobrychis supina* (Vill.) DC. (Fabaceae) BCN52589	Trepadella, trepadella borda	MO	Flower	Agrosilvopastoral management: agricultural/horticultural	1
*Onobrychis viciifolia* Scop. (Fabaceae) BCN26745	Esparceta, trepadella	MO	Flower	Agrosilvopastoral management: agricultural/horticultural	1
*Ononis spinosa* L. (Fabaceae) BCN50770	Gavó	AE	Stem	Fuel obtaining: firewood	1
*Ononis spinosa* L. subsp. *antiquorum* (L.) Briq. (Fabaceae) BCN62318	Augó, ugó	BI	Fruit	Other informations: undesirable actions	1
*Ophrys speculum* Link (Orchidaceae) BCN533	Orquídia, sabateta de bon Jesús	BI	Aerial part	Ludic Ornamental: bouquet elaborations	2
*Opuntia maxima* Mill. (Cactaceae) BCN46078	Fulla de figa de moro (cladode), fulla de moro, fulla de moro (cladode), fulla de figuera de moro (cladode), figa de moro (fruit), figuera de moro, figuera de pic	BI	Cladode. Fruit. Leaf. Leaf juice. Whole plant,	Agrosilvopastoral management: tools for agricultural practices Artisanal: cookware elaboration Cosmetic Domestic: for help at home Ludic Other informations: excipient or adjuvant in medicinal or food preparations	19
*Origanum majorana* L. (Lamiaceae) BCN95545	Marduix, morduix, morduixera	BI, MO, RI	Aerial part. Leaf. Whole plant	Agrosilvopastoral management: agricultural/horticultural Cosmetic Folk oral literature Other informations: ecological information	8
*Origanum vulgare* L. (Lamiaceae) BCN24938	Orenga, clements	AE, BI, RI	Aerial part. Flower. Whole plant	Domestic: air freshener Domestic: for help at home Folk oral literature Other informations: harvesting and/or selling Repellent	16
*Orobanche crenata* Forsk. (Orobanchaceae) BCN29743	Frare	AE	Whole plant	Other informations: ecological information	1
*Oryza sativa* L. (Poaceae) BCN30000	Arròs	AE, BI, MO	Bran. Not reported. fruit. Stem	Agrosilvopastoral management: agricultural/horticultural Domestic: for help at home Magic and religious beliefs and practices Other informations: excipient or adjuvant in medicinal or food preparations	4
*Oryzopsis miliacea* (L.) Asch. & Graebn. (Poaceae) BCN52590	Fenal no vertader, herba prima	MO	Aerial part	Ornamental: bouquet elaboration	2
*Osyris alba* L. (Santalaceae) BCN100425	Assot, ginestra	BI	Aerial part	Other informations: ecological information	1
*Oxalis articulata* Savigny (Oxalidaceae) BCN31290	Trèvol	AE	Leaf. Whole plant	Magic and religious beliefs and practices Other informations: ecological information	5
*Oxalis pes-caprae* L. (Oxalidaceae) BCN95553	Vinagrella, trèvol de jardí	BI	Whole plant	Agrosilvopastoral management: weeds Folk oral literature Other informations: ecological information	5
*Paeonia mascula* (L.) Mill. subsp. *cambessedesii* (Willk.) O.Bolòs & Vigo (Paeoniaceae) BCN64801	Paloni de boca, peònia	BI	Whole plant	Folk oral literature Ornamental: gardening	3
*Paliurus spina-christi* Mill. (Rhamnaceae) BCN29902	Espinavessa	AE	Root. Stem	Artisanal: cane elaboration Domestic: for help at home Magic and religious beliefs and practices	6
*Pancratium maritimum* L. (Amaryllidaceae) BCN13292	Lliri	AE	Aerial part	Ornamental: bouquet elaboration	2
*Papaver rhoeas* L. (Papaveraceae) BCN29903	Coquelicot (French), gall, gall-gallaret, gallaret, gallito (Spanish), quiquiriquic, roella, rogella, rosella, rosella de camp, rovella	AE, BI, MO, RI	Corolla. Floral bud. Flower. Fruit. Whole plant.	Cosmetic Ludic Magic and religious beliefs and practices Ornamental: bouquet elaboration Other informations: ecological information	29
*Papaver somniferum* L. (Papaveraceae) BCN24941	Cascall, dormidera (Spanish)	BI, RI	Aerial part. Flower. Fruit. Whole plant	Ornamental: bouquet elaboration Ornamental: gardening Other informations: ecological information Other informations: appreciations on plant or product properties	6
*Papaver somniferum* L. subsp. *setigerum* (DC.) Arcang. (Papaveraceae) BCN46071	Cascall, cascai, dormidera (Spanish), roella	AE, BI	Aerial part. Flower. Fruit. Latex. Whole plant	Ornamental: bouquet elaboration Ornamental: gardening Other informations: ecological information	8
*Papaver somniferum* L. subsp. *somniferum* (Papaveraceae) BCN126627	Cascall, dormidera (Spanish), herba queixalera	MO	Whole plant	Ornamental: gardening	5
*Paradisea liliastrum* (L.) Bertol. (Liliaceae) BCN27270	Lliri blanc	RI	Aerial part	Ornamental: bouquet elaboration	1
*Parietaria officinalis* L. subsp. *judaica* (L.) Bég. (Urticaceae) BCN29745	Cama-roja, herba mollera, molla roquera, mollera, mollera roquera, mollerusa, morella, morella roquera, morera roquera, roca mollera, parietària	AE, BI, MO	Aerial part. Leaf. Not reported. Stem. Whole plant	Agrosilvopastoral management: weeds Ludic Magic and religious beliefs and practices Other informations: ecological information Other informations: use not remembered	16
*Passiflora caerulea* L. (Passifloraceae) BCN29747	Claus de l’infern, passiflora	AE	Aerial part	Ornamental: bouquet elaboration	1
*Pelargonium capitatum* (L.) L’Hér. (Geraninaceae) BCN72719	Gerani d’olor, vauma rosa	BI	Aerial part. Flower. Whole plant	Agrosilvopastoral management: tools for agricultural practices Ornamental: bouquet elaboration Ornamental: gardening Other informations: ecological information Repellent	8
*Pelargonium* sp. (Geraniaceae).	Gerani	BI	Aerial part	Domestic: air freshener Other informations: ecological information	2
*Petroselinum crispum* (Mill.) Fuss (Apiaceae) BCN29905	Julivert	AE, BI RI	Aerial part. Leaf. Stem. Whole plant	Agrosilvopastoral management: agricultural/horticultural Cosmetic Folk oral literature Other informations: ecological information	7
*Phagnalon saxatile* (L.) Cass. (Asteraceae) BCN95555	Herba de capxerigany	BI	Whole plant	Other informations	1
*Phaseolus vulgaris* L. (Fabaceae) BCN46837	Mongeta, mongeta seca	AE, BI	Seed. Whole plant	Agrosilvopastoral management: agricultural/horticultural Ludic	2
*Philadelphus coronarius* L. (Hydrangeaceae) BCN27261	Xeringuilla, xeringuillo	BI, MO, RI	Aerial part. Flower. Whole plant	Magic and religious beliefs and practices Ornamental: bouquet elaboration Ornamental: gardening Timber: construction materials production	12
*Phillyrea angustifolia* L. (Oleaceae) BCN126631	Mat, mata	MO	Stem	Agrosilvopastoral management: tools for agricultural practices	1
*Phlomis italica* L. (Lamiaceae) BCN64879	Estepa blenera	BI	Leaf. Not reported	Cosmetic Domestic: for help at home	2
*Phoenix canariensis* Chabaud (Arecaceae) BCN23835	Fasser, dàtil (fruit), palmera, paumera	BI	Leaf. Whole plant	Artisanal: basketry Magic and religious beliefs and practices Ornamental: bouquet elaboration Ornamental: gardening	6
*Phoenix dactylifera* L. (Arecaceae) BCN51783	Fasser, dàtil (fruit), palmera, pauma, paumera	BI, MO	Aerial part. Leaf, Thorn. Whole plant	Artisanal: basketry Artisanal: cookware elaboration Magic and religious beliefs and practices Ornamental: bouquet elaboration Other informations: ecological information	9
*Phragmites australis* (Cav.) Trin. ex Steud. (Poaceae) BCN88033	Canyet, canyisso	BI	Not reported	Textile: fibre or cloth elaboration	1
*Phyllitis scolopendrium* (L.) Newman (Aspleniaceae) BCN29906	Herba melsera, melsera	MO	Frond	Ornamental: gardening	1
*Phyllostachys aurea* Rivière & C.Rivière (Poaceae) BCN30002	Canya americana, canya de bambú, canya forastera, bambú	AE, BI	Stem. Whole plant	Agrosilvopastoral management: tools for agricultural practices Agrosilvopastoral management: for fishing Artisanal: musical instrument elaboration Other informations: ecological information	14
*Phyllostachys flexuosa* Rivière & C.Rivière (Poaceae) BCN30001	Canya americana negra	AE	Stem	Agrosilvopastoral management: for fishing	1
*Physalis alkekengi* L. (Solanaceae) BCN31293	Alquequengi, bossa, bossa vermella, fanalet, pebrot	AE, RI, MO	Aerial part. Fruit. Whole plant	Ornamental: bouquet elaboration Ornamental: gardening	3
*Phytolacca americana* L. (Phytolaccaceae) BCN81961	Fitolaca	MO	Fruit	Ludic	1
*Picea abies* (L.) H.Karst. (Pinaceae) BCN36717	Avet, pivet	MO	Whole plant	Ornamental: gardening	2
*Picris hieracioides* Sibth. & Sm. (Asteraceae) BCN49161	Gafallosa, pedaç	MO	Leaf	Ludic	2
*Pimpinella anisum* L. (Apiaceae) BCN47278	Matafaluga	AE	Fruit	Domestic: air freshener	1
*Pinus halepensis* Mill. (Pinaceae) BCN29826	Pi, pi bord, pinya (cone), reïna (resin)	AE, BI, MO	Aerial part. Bark. Leaf. Not reported. Resin. Stem. Whole plant	Agrosilvopastoral management: forestry Artisanal: broom elaboration Artisanal: cookware elaboration Artisanal: footwear elaboration Artisanal: instrument elaboration Artisanal: musical instrument elaboration Artisanal: tannery Domestic: air freshener Domestic: for help at home Fuel obtaining: charcoal Fuel obtaining: firewood Magic and religious beliefs and practices Ornamental: bouquet elaboration Ornamental: gardening Other informations: ecological information Other informations: harvesting and/or selling Other informations: use not remembered Textile: dyer Timber Timber: boat manufacturing Timber: wheeled vehicles manufacturing Timber: furniture manufacturing Timber: construction materials production	51
*Pinus pinaster* Aiton (Pinaceae) BCN36559	Pi, pi bord, pi gallec, pinastre	AE, MO	Stem	Agrosilvopastoral management: honey obtaining Other informations: use not remembered Timber: boat manufacturing	3
*Pinus pinea* L. (Pinaceae) BCN26751	Pi, pi de llei, pi pinyer, pi pinyoner, pinyó (seed)	AE, BI, MO	Bark. Leaf. Seed. Stem. Whole plant	Artisanal: footwear elaboration Folk oral literature Fuel obtaining: firewood Magic and religious beliefs and practices Ornamental: bouquet elaboration Ornamental: gardening Textile: dyer Timber Timber: boat manufacturing Timber: wheeled vehicles manufacturing Timber: construction materials production	18
*Pinus* sp. (Pinaceae)	Pi, pinya (cone), reïna, trementina (elaborated product)	AE, BI, MO	Aerial part. Bark, Flower. Leaf. Resin. Stem. Whole plant. Cortical parenchyma	Agrosilvopastoral management: forestry Agrosilvopastoral management: honey obtaining Cosmetic Domestic: for help at home Folk oral literature Fuel obtaining: charcoal Fuel obtaining: firewood Magic and religious beliefs and practices Other informations: harvesting and/or selling Repellent Smoking Textile: dyer	26
*Pinus sylvestris* L. (Pinaceae) BCN46070	Pi, pi bord, pi rajolet, pi roig, trementina (elaborated product), pega (elaborated product), pega negra (elaborated product)	AE, MO, RI	Cone. Stem. Whole plant	Artisanal: footwear elaboration Fuel obtaining: firewood Other informations: ecological information Timber: boat manufacturing Timber: construction materials production	12
*Piper nigrum* L. (Piperaceae) BCN47277	Prebe bo, prebe bo blanc, prebe negre	BI	Seed	Agrosilvopastoral management: for pig slaughter	1
*Pistacia lentiscus* L. (Anacardiaceae) BCN29907	Llentriscle, llentrisca, mat, mata, triquell	AE, BI, MO	Aerial part. Fruit juice. Leaf. Not reported. Resin. Stem.	Agrosilvopastoral management: tools for agricultural practices Artisanal: basketry Artisanal: musical instrument elaboration Domestic: air freshener Domestic: for help at home Fuel obtaining: charcoal Magic and religious beliefs and practices Ornamental: bouquet elaboration Other informations: use not remembered Repellent Timber	33
*Pistacia terebinthus* L. (Anacardiaceae) BCN30004	Noguerola	AE	Resin	Domestic: paint elaboration	1
*Pistacia vera* L. (Anacardiaceae) BCN-E-87	Pistatxo	MO	Whole plant	Ornamental: gardening	1
*Pisum sativum* L. subsp. *sativum* (Fabaceae) BCN32140	Estiragassó, pèsol, xítxero	BI	Whole plant	Agrosilvopastoral management: agricultural/horticultural Other informations: ecological information	3
*Plantago afra* L. (Plantaginaceae) BCN 95568	Herba de puça	BI	Aerial part. Seed	Cosmetic	3
*Plantago coronopus* L. subsp. *coronopus* (Plantaginaceae) BCN29908	Cua de rata	AE	Leaf	Magic and religious beliefs and practices	2
*Plantago lagopus* L. (Plantaginaceae) BCN98975	Herba de cinc venes	BI	Not reported	Other informations: ecological information	1
*Plantago lanceolata* L. (Plantaginaceae) BCN24949	Herba de cinc venes, llantén (Spanish), plantatge, plantatge de fulla estreta, plantatge estret, plantatge llarg, plantago	BI, MO, RI	Aerial part. Leaf. Whole plant	Domestic: for help at home Ludic Other informations: ecological information	6
*Plantago major* L. (Plantaginaceae) BCN24950	Llantén (Spanish), plantatge, plantatge ample, plantatge rodó	BI, RI	Leaf	Ludic Other informations: ecological information	2
*Platanus* ×*hispanica* Mill. ex Münchh. (Platanaceae) BCN31294	Plàtan, plataner, platero	AE, BI, MO	Bark. Leaf. Stem	Agrosilvopastoral management: tools for agricultural practices Domestic: for help at home Magic and religious beliefs and practices Timber Timber: boat manufacturing Timber: furniture manufacturing	10
*Plectanthus* sp. (Lamiaceae)	Encens, planta dels doblers	BI	Whole plant	Ornamental: gardening	2
*Poinsettia pulcherrima* (Wild.) Graham (Euphorbiaceae) BCN48713	Nadala	AE	Whole plant	Magic and religious beliefs and practices	1
*Polygonatum odoratum* (Mill.) Druce (Liliaceae) BCN56044	Arracadetes	MO	Aerial part	Ornamental: bouquet elaboration	1
*Polygonum aviculare* L. (Polygonaceae) BCN32136	Estiravelles, triavella, traspassacamins	MO	Whole plant	Folk oral literature	1
*Polypodium vulgare* L. (Polypodiaceae) BCN32134	Falzia, foguera borda, herba de les gallines, herba felera	MO, RI	Frond. Not reported	Folk oral literature Ornamental: bouquet elaboration	2
*Polystichum setiferum* (Forssk.) Moore ex Woyn. (Dryopteridaceae) BCN126634	Foguera, foguera de jardí, foguera mosquera	MO	Frond	Magic and religious beliefs and practices Ornamental: bouquet elaboration	20
*Populus alba* L. (Salicaceae) BCN29751	Arbre, arbre poll de fusta blanca, alba, arbre blanc	AE, MO	Stem	Agrosilvopastoral management: tools for agricultural practices Artisanal: footwear elaboration Ludic Timber	8
*Populus nigra* L. (Salicaceae) BCN29753	Arbre canadà, arbre poll de fulla fosca, arbre poll, poll, polla, pollancre, pollangre	AE, BI, MO, RI	Stem	Agrosilvopastoral management: tools for agricultural practices Artisanal: footwear elaboration Domestic: for help at home Ludic Other informations Timber Timber: furniture manufacturing	15
*Populus* sp. (Salicaceae)	Poll, pollancre	BI, RI	Stem	Other informations: use not remembered Timber: furniture manufacturing	3
*Populus tremula* L. (Salicaceae) BCN46836	Trèmol	AE, MO	Stem	Artisanal: cane elaboration Artisanal: cookware elaboration Timber	6
*Populus* × *canadensis* Moench (Salicaceae) BCN126637	Poll, polla, pollancre bordil	BI, MO	Stem. Whole plant	Artisanal: cookware elaboration Ornamental: gardening Timber Timber: construction materials production	4
*Portulaca oleracea* L. (Portulacaceae) BCN46835	Enciam de patena, verdelaga, verdolaga	AE, BI	Whole plant	Folk oral literature Ornamental: gardening Other informations: ecological information	3
*Posidonia oceanica* (L.) Delile (Posidoniaceae) BCN103134	Alga, bolla de la mar (decomposed stem and leaf), posidònia	BI	Aerial part. Leaf. Stem. Whole plant	Agrosilvopastoral management: tools for agricultural practices Artisanal: musical instrument elaboration Domestic: for help at home Other informations: harvesting and/or selling Textile: textile padding	18
*Potentilla reptans* L. (Rosaceae) BCN29754	Gram corquim	AE	Whole plant	Other informations: ecological information	1
*Primula veris* L. (Primulaceae) BCN27280	Cucuts	MO, RI	Aerial part. Flower	Magic and religious beliefs and practices Ornamental: bouquet elaboration	6
*Primula veris* L. subsp. *columnae* (Ten.) Maire & Petitm. (Primulaceae) BCN29755	Puput	AE	Aerial part	Ornamental: bouquet elaboration	1
*Prunella grandiflora* (L.) Scholler (Lamiaceae) BCN24956	Herba del traïdor, xuclaabella	RI	Aerial part	Agrosilvopastoral management: honey obtaining	1
*Prunella grandiflora* (L.) Scholler subsp. *pyrenaica* (Gren. & Godr.) A. & O.Bolòs (Lamiaceae) BCN29757	Herba de la mel, xuclamel	AE, MO	Inflorescence. Whole plant	Agrosilvopastoral management: honey obtaining Ornamental: bouquet elaboration Other informations: ecological information	9
*Prunella vulgaris* L. (Lamiaceae) BCN29759	Herba del traïdor	AE	Flower	Smoking	1
*Prunus armeniaca* L. (Rosaceae) BCN48712	Albercoc (fruit), albercoquer	BI	Endocarp. Fruit. Stem	Artisanal: musical instrument elaboration Folk oral literature Fuel obtaining: charcoal Fuel obtaining: firewood Ludic Other informations: appreciations on plant or product properties Timber: furniture manufacturing	7
*Prunus avium* (L.) L. (Rosaceae) BCN29827	Cirera (fruit), cirerer	AE, BI, MO	Fruit. Resin. Seed. Stem. Whole plant	Artisanal: basketry Artisanal: cane elaboration Domestic: for help at home Folk oral literature Ludic Other informations: appreciations on plant or product properties Timber Timber: furniture manufacturing	17
*Prunus avium* (L.) L. var. *sylvestris* Dierb. (Rosaceae) BCN60586	Cirerer	MO	Flower. Whole plant	Agrosilvopastoral management: agricultural/horticultural	3
*Prunus domestica* L. (Rosaceae) BCN46834	Pruna (fruit), prunera	BI	Aerial part. Fruit. Whole plant	Agrosilvopastoral management: agricultural/horticultural Folk oral literature Ludic Other informations: ecological information	4
*Prunus dulcis* (Mill.) D.A.Webb. (Rosaceae) BCN46833	Ametla (fruit), ametler, ametller	AE, BI, MO	Aerial part. Endocarp. Epicarp. Flower. Fruit. Fruit juice. Mesocarp. Resin. Seed. Stem. Whole plant	Agrosilvopastoral management: agricultural/horticultural Artisanal: musical instrument elaboration Cosmetic Domestic: for help at home Folk oral literature Fuel obtaining: charcoal Fuel obtaining: firewood Ornamental: bouquet elaboration Other informations: use not remembered Timber Timber: boat manufacturing Timber: furniture manufacturing	43
*Prunus persica* (L.) Batsch (Rosaceae) BCN52783	Melicotó (fruit), melicotoner, préssec (fruit), presseguer	BI, MO	Flower. Whole plant	Agrosilvopastoral management: agricultural/horticultural Other informations: ecological information	2
*Prunus spinosa* L. (Rosaceae) BCN126640	Aranyoner, arç negre, prunyoner	BI, MO	Stem. Whole plant	Agrosilvopastoral management: agricultural/horticultural Other informations: ecological information Timber	4
*Pseudotsuga menziesii* (Mirb.) Franco (Pinaceae) BCN36632	Douglas, pi Douglas	MO	Leaf. Whole plant	Magic and religious beliefs and practices Timber	4
*Psoralea bituminosa* L. (Fabaceae) BCN32133	Cabrulla, calabrunya	MO	Flower	Magic and religious beliefs and practices	1
*Pteridium aquilinum* (L.) Kuhn (Dennstaedtiaceae) BCN46068	Falguera, falguera de Lluc, helecho (Spanish), mata falguera	AE, BI, MO, RI	Aerial part. Frond. Whole plant	Agrosilvopastoral management: agricultural/horticultural Domestic: for help at home Folk oral literature Magic and religious beliefs and practices Ornamental: bouquet elaboration Ornamental: gardening Other informations: use not remembered	34
*Punica granatum* L. (Lythraceae) BCN29764	Magrana (fruit), magraner	AE, BI, MO	Flower. Fruit. Stem. Whole plant	Agrosilvopastoral management: for pig slaughter Artisanal: cane elaboration Artisanal: cookware elaboration Artisanal: musical instrument elaboration Domestic: for help at home Folk oral literature Ludic Magic and religious beliefs and practices Ornamental: bouquet elaboration Ornamental: gardening Other informations: ecological information Textile: dyer	16
*Pyracantha coccinea* M.Roem. (Rosaceae) BCN52591	Cartegus	MO	Whole plant	Ornamental: gardening	1
*Pyrus communis* L. subsp. *communis* (Rosaceae) BCN46831	Pera (fruit), perera	AE, BI, MO	Flower. Stem. Whole plant	Agrosilvopastoral management: agricultural/horticultural Artisanal: musical instrument elaboration Other informations: ecological information	3
*Pyrus malus* L. subsp. *malus* (Rosaceae) BCN24961	Pomera borda	RI	Aerial part	Agrosilvopastoral management: agricultural/horticultural	1
*Pyrus malus* L. subsp. *mitis* (Wallr.) O.Bolòs & J.Vigo (Rosaceae) BCN46830	Poma (fruit), poma bellesa de Roma (fruit), poma de bosc (fruit), poma del ciri (fruit), poma estarqui (fruit), poma fadrineta (fruit), poma goldén (fruit), poma manyaga (fruit), poma reneta (fruit), pomera, pomera borda, vinagre de poma (elaborated product)	AE, BI, MO	Fruit. Whole plant	Agrosilvopastoral management: agricultural/horticultural Artisanal: cane elaboration Domestic: air freshener Other informations: ecological information	8
*Quercus alba* L. (Fagaceae) BCN60896	Roure americà	AE	Stem	Domestic: for help at home	1
*Quercus coccifera* L. subsp. *coccifera* (Fagaceae) BCN29765	Garric, garriga, garrolla	AE	Stem. Whole plant	Artisanal: broom elaboration Other informations: ecological information	2
*Quercus humilis* Mill. (Fagaceae) BCN32132	Roure	MO	Gall. Stem. Whole plant	Fuel obtaining: charcoal Ludic Timber	4
*Quercus ilex* L. (Fagaceae) BCN24963	Aglà (fruit), alzina, aulina	BI, RI	Bark. Flower. Leaf. Stem. Whole plant	Agrosilvopastoral management: honey obtaining Agrosilvopastoral management: tools for agricultural practices Artisanal Artisanal: tannery Cosmetic Folk oral literature Fuel obtaining: firewood Ludic Magic and religious beliefs and practices Ornamental: gardening Other informations: ecological information Other informations: use not remembered Textile: dyer Timber Timber: boat manufacturing Timber: furniture manufacturing	31
*Quercus ilex* L. subsp. *ilex* (Fagaceae) BCN29932	Aglà (fruit), alzina, roure	AE, BI, MO	Bark. Leaf. Stem	Agrosilvopastoral management: tools for agricultural practices Agrosilvopastoral management: for pig slaughter Artisanal: broom elaboration Artisanal: cane elaboration Artisanal: cookware elaboration Domestic: for help at home Folk oral literature Fuel obtaining: charcoal Fuel obtaining: firewood Ludic Magic and religious beliefs and practices Ornamental: gardening Timber: boat manufacturing Timber: wheeled vehicles manufacturing	52
*Quercus petraea* (Matt.) Liebl. (Fagaceae) BCN29829	Aglà (fruit), alzina, roure, roure francès, roure hongarès	AE, MO, RI	Fruit. Gall. Stem. Whole plant. Cortical parenchyma	Agrosilvopastoral management: tools for agricultural practices Fuel obtaining: charcoal Fuel obtaining: firewood Ludic Other informations: ecological information Timber: boat manufacturing Timber: furniture manufacturing Timber: construction materials production	16
*Quercus pubescens* Willd. (Fagaceae) BCN30007	Roure	AE	Stem	Agrosilvopastoral management: tools for agricultural practices	1
*Quercus suber* L. (Fagaceae) BCN46829	Alzina surera, suro	AE, MO	Bark	Agrosilvopastoral management: tools for agricultural practices Agrosilvopastoral management: for fishing Domestic: for help at home Folk oral literature Ludic Other informations: use not remembered Textile: dyer Textile: textile padding	9
*Ranunculus asiaticus* L. (Ranunculaceae) BCN65283	Francesilla	BI	Aerial part. Whole plant	Ornamental: bouquet elaboration Ornamental: gardening	3
*Raphanus raphanistrum* L. (Brassicaceae) BCN30042	Ravenissa	BI	Whole plant	Agrosilvopastoral management: agricultural/horticultural	1
*Raphia* sp. (Arecaceae)	Ràfia	BI	Leaf	Artisanal: basketry	1
*Rhamnus alaternus* L. (Rhamnaceae) BCN29769	Aladern, llampúdol, llampudoler, mata	AE, BI, MO	Aerial part. Stem. Whole plant	Artisanal: broom elaboration Artisanal: cookware elaboration Artisanal: smoking pipe elaboration Fuel obtaining: charcoal Ornamental: bouquet elaboration Ornamental: gardening Timber Timber: furniture manufacturing	12
*Rhamnus ludovici-salvatoris* Chodat (Rhamnaceae) BCN30057	Aladern, llampúdol	BI	Whole plant	Ornamental: gardening	1
*Rhododendron ferrugineum* L. (Ericaceae) BCN24969	Gafet, neret	RI	Aerial part. Whole plant	Ornamental: bouquet elaboration Other informations: ecological information	3
*Ricinus communis* L. (Euphorbiaceae) BCN46089	Cagamutxo, oli de ricí (elaborated product), recino (Spanish), resiner, ricino (Spanish)	AE, BI	Seed. Seed oil	Magic and religious beliefs and practices Other informations: purchased in commerce	13
*Robinia pseudoacacia* L. (Fabaceae) BCN31298	Acàcia, escàcia	AE, BI, MO, RI	Flower. Not reported. Stem	Agrosilvopastoral management: tools for agricultural practices Domestic: for help at home Magic and religious beliefs and practices Timber: boat manufacturing Timber: wheeled vehicles manufacturing Timber: furniture manufacturing Timber: construction materials production	11
*Rorippa nasturtium-aquaticum* (L.) Hyek (Brassicaceae) BCN29771	Créixec, creixen, créixol, créssec	BI	Aerial part	Other informations: appreciations on plant or product properties	2
*Rosa alba* L. (Rosaceae) BCN400041	Roser blanc	BI	Whole plant	Ornamental: gardening	1
*Rosa canina* L. (Rosaceae) BCN29772	Gavarrera, roser bord, roser de bosc, roser de pastor, roser silvestre	AE, MO	Aerial part. Fruit. Stem. Whole plant	Agrosilvopastoral management: tools for agricultural practices Artisanal: smoking pipe elaboration Ludic Ornamental: bouquet elaboration	6
*Rosa gallica* L. (Rosaceae) BCN24972	Roser, roser de marge	MO, RI	Flower. Whole plant	Folk oral literature Magic and religious beliefs and practices Ornamental: bouquet elaboration	7
*Rosa* sp. (Rosaceae)	Roser, roser de jardí	AE, BI, MO, RI	Corolla. Flower. Leaf. Whole plant	Agrosilvopastoral management: tools for agricultural practices Cosmetic Domestic: air freshener Folk oral literature Magic and religious beliefs and practices Ornamental: bouquet elaboration Ornamental: gardening Other informations: ecological informations	24
*Rosa tomentosa* Sm. (Rosaceae) BCN24973	Grataculs, roser, roser bord, roser de bosc	RI	Fruit. Whole plant	Ludic Ornamental: gardening	2
*Rosa centifolia* L. (Rosaceae) BCN126648	Roser, roser de marge	MO	Flower	Magic and religious beliefs and practices Other informations	2
*Rosmarinus officinalis* L. (Lamiaceae) BCN29937	Romaní	AE, BI, MO, RI	Aerial part. Flower. Stem. Whole plant	Agrosilvopastoral management: weeds Cosmetic Domestic: air freshener Folk oral literature Magic and religious beliefs and practices Other informations: ecological information Repellent	43
*Rubus ulmifolius* Schott (Rosaceae) BCN32130	Barder, batzer, móra (fruit), romeguer, romeguera	BI, MO	Flower. Whole plant. Young shoot	Agrosilvopastoral management: honey obtaining Folk oral literature Other informations: ecological information	10
*Rumex acetosa* L. subsp. *acetosa* (Polygonaceae) BCN29775	Llengua de bou	AE	Aerial part	Ornamental: bouquet elaboration	1
*Rumex crispus* L. (Polygonaceae) BCN126650	Cama-roja, paradella, Santa Maria, vinagrella	MO	Fruit	Magic and religious beliefs and practices	3
*Rumex* sp. (Polygonaceae)	Llengua de bou	RI	Leaf	Ludic	1
*Ruscus aculeatus* L. (Asparagaceae) BCN29939	Bolleta del bon pastor, bolleta de Nadal, boix marí, cirerer de Betlem, gallaranc, galzeran	AE, BI, MO, RI	Aerial part. Stem. Whole plant	Agrosilvopastoral management: for fishing Magic and religious beliefs and practices Ornamental: bouquet elaboration Ornamental: gardening Other informations: use not remembered	20
*Ruta chalepensis* L. (Rutaceae) BCN29940	Abrecaminos (Spanish), ruda	AE, BI, RI	Aerial part. Not reported. Stem. Whole plant	Agrosilvopastoral management: tools for agricultural practices Cosmetic Domestic: air freshener Folk oral literature Magic and religious beliefs and practices Ornamental: bouquet elaboration Other informations: ecological information Other informations: appreciations on plant or product properties Repellent	37
*Ruta chalepensis* L. subsp. *chalepensis* (Rutaceae) BCN32128	Ruda	MO	Whole plant	Folk oral literature	6
*Ruta graveolens* L. (Rutaceae) BCN29776	Ruda	BI	Aerial part	Magic and religious beliefs and practices	5
*Saccharum officinarum* L. (Poaceae) BCN50771	Canya de sucre, canyamel, melassa (elaborated product)	BI	Stem	Agrosilvopastoral management: tools for agricultural practices	1
*Salix alba* L. (Salicaceae) BCN29777	Saula, saule	AE, RI	Stem	Artisanal: broom elaboration Artisanal: footwear elaboration Agrosilvopastoral management: for fishing Ornamental: bouquet elaboration Other informations: ecological information	6
*Salix alba* L. subsp. *alba* (Salicaceae) BCN56012	Saula, saule, sàlic	MO, RI	Flower. Stem	Agrosilvopastoral management: tools for agricultural practices Artisanal: footwear elaboration Timber: wheeled vehicles manufacturing	8
*Salix atrocinerea* Brot. (Salicaceae) BCN39996	Gatell	AE	Whole plant	Other informations: ecological information	1
*Salix babylonica* L. (Salicaceae) BCN50772	Desmai	AE, MO	Stem. Whole plant	Artisanal: basketry Ornamental: gardening	4
*Salix caprea* L. (Salicaceae) BCN46073	Gat saule, gatells, salze, saule	AE, RI	Aerial part. Stem	Ornamental: bouquet elaboration	6
*Salix cinerea* L. subsp. *oleifolia* Macreight (Salicaceae) BCN126656	Gatell	MO	Aerial part. Stem	Artisanal: basketry Artisanal: cane elaboration Ornamental: bouquet elaboration Timber	11
*Salix elaeagnos* Scop. subsp. *angustifolia* (Cariot) Rech.f. (Salicaceae) BCN31304	Salits, salitres, sàlix, sarga, vergatera	AE, MO	Stem	Artisanal: basketry	7
*Salix* ×*fragilis* L. (Salicaceae) BCN31305	Saula, saule, salze dels vims, saula vimera, vim, vimenera, vímet	AE, MO	Stem	Artisanal: basketry Artisanal: broom elaboration Artisanal: footwear elaboration Agrosilvopastoral management: for fishing Ludic	31
*Salsola kali* L. subsp. *ruthenica* (Iljin) Soó (Amaranthaceae) BCN42985	Saleret, sosa	BI	Not reported	Cosmetic Domestic: paint elaboration	2
*Salsola soda* L. (Amaranthaceae) BCN42984	Saleret	BI	Not reported	Domestic: paint elaboration	1
*Salsola vermiculata* L. (Amaranthaceae) BCN83712	Barrilla, barrillera	BI	Not reported	Other informations: ecological information	1
*Salvia microphylla* Kunth (Lamiaceae) BCN126657	Gessamí, menta, menta de la reina, menta piperita, menta romana, menta trapassera, ormí, senyorida	BI, MO	Flower. Whole plant	Agrosilvopastoral management: honey obtaining Ludic Ornamental: gardening	7
*Salvia officinalis* L. (Lamiaceae) BCN95591	Salvi, sàlvia	BI	Aerial part. Leaf. Not reported. Whole plant	Agrosilvopastoral management: tools for agricultural practices Cosmetic Domestic: for help at home Folk oral literature Ornamental: gardening Other informations: ecological information Other informations: appreciations on plant or product properties	17
*Salvia officinalis* L. subsp. *lavandulifolia* (Vahl) Gams (Lamiaceae) BCN1674	Sàlvia, sàlvia femella, sàlvia mascle	MO	Whole plant	Folk oral literature	1
*Salvia* sp. (Lamiaceae)	Sàlvia	BI	Whole plant	Other informations: ecological informations	1
*Salvia verbenaca* L. (Lamiaceae) BCN29942	Tàrrec, tàrrega	BI, RI	Leaf. Not reported	Smoking	5
*Sambucus nigra* L. (Adoxaceae) BCN29943	Sabuc, sabuquer, saüc, saüquer	AE, BI, RI	Flower. Stem. Whole plant	Agrosilvopastoral management: tools for agricultural practices Domestic: air freshener Ludic Magic and religious beliefs and practices Other informations: ecological information Other informations: harvesting and/or selling	9
*Santolina chamaecyparissus* L. (Asteraceae) BCN31526	Camamilla de botó, camamilla de botó groc, camamilla de l’hort, espernallac	MO, RI	Aerial part. Inflorescence	Cosmetic Domestic: air freshener Folk oral literature Ornamental: bouquet elaboration	4
*Santolina chamaecyparissus* L. subsp. *chamaecyparissus* (Asteraceae) BCN29782	Camamilla, camamilla de muntanya	AE	Aerial part. Inflorescence	Cosmetic Ornamental: bouquet elaboration	3
*Santolina chamaecyparissus* L. subsp. *magonica* O.Bolòs & al. (Asteraceae) BCN-E-186	Camamil · la	BI	Aerial part. Inflorescence. Whole plant	Agrosilvopastoral management: tools for agricultural practices Cosmetic Domestic: air freshener Folk oral literature Magic and religious beliefs and practices Other informations: ecological information Repellent	20
*Saponaria officinalis* L. (Caryophyllaceae) BCN29783	Herba del sabó, herba ensabonera, herba sabonera, sabó, sabó de gitana	AE, MO, RI	Aerial part. Flower	Cosmetic Domestic: for help at home	11
*Sarothamnus arboreus* (Desf.) Webb subsp. *catalaunicu*s (Webb) C.Vic (Fabaceae) BCN29784	Ginesta borda, ginestell, ginestó	AE	Aerial part. Stem. Whole plant	Agrosilvopastoral management: tools for agricultural practices Ornamental: bouquet elaboration Other informations: ecological information	3
*Sarothamnus scoparius* (L.) Wimm. ex Koch (Fabaceae) BCN24988	Ginesta, ginesta prima, ginestell, gódua, lliroia	MO, RI	Aerial part. Flower	Artisanal: broom elaboration Domestic: for help at home Fuel obtaining: charcoal Magic and religious beliefs and practices Ornamental: bouquet elaboration	22
*Satureja calamintha* (L.) Scheele (Lamiaceae) BCN109263	Menta borda, rementerola	MO	Flower. Leaf	Agrosilvopastoral management: agricultural/horticultural Domestic: for help at home	2
*Satureja calamintha* (L.) Scheele subsp. *glandulosa* (Req.) Gams (Lamiaceae) BCN29785	Clements	AE	Stem	Domestic: for help at home	1
*Satureja hortensis* L. (Lamiaceae) BCN29945	Senyorida	BI	Whole plant	Other informations: use not remembered	1
*Saxifraga longifolia* Lapeyr. (Saxifragaceae) BCN54656	Corona de rei	RI	Whole plant	Ornamental: bouquet elaboration	1
*Saxifraga vayredana* Luizet (Saxifragaceae) BCN95599	Herba de Sant Segimon	MO	Aerial part. Whole plant	Folk oral literature Other informations: ecological information Smoking	11
*Scabiosa atropurpurea* L. (Dipsacaceae) BCN29947	Escabiosa, escabriosa, escapiosa, herba escapriosa	AE, BI	Not reported. Whole plant	Other informations: ecological information	3
*Schefflera arborea* (L.) M.Gómez (Araliaceae) BCN129084	Xeflera	BI	Whole plant	Ornamental: gardening	1
*Schinus molle* L. (Anacardiaceae) BCN46086	Pebre verd	AE	Aerial part	Ornamental: bouquet elaboration	1
*Scirpus holoschoenus* L. (Cyperaceae) BCN29789	Jonc	AE, BI, MO	Aerial part. Stem	Agrosilvopastoral management: tools for agricultural practices Agrosilvopastoral management: for fishing Agrosilvopastoral management: for hunting Agrosilvopastoral management: for pig slaughter Artisanal: basketry Domestic: for help at home Other informations: ecological information Textile: fibre or cloth elaboration	42
*Scolymus hispanicus* L. (Asteraceae) BCN30008	Card cadalina, card catalina, card de carxofa, card de cadernina, card cadernina, card verd	BI	Whole plant	Agrosilvopastoral management: for pig slaughter Folk oral literature Other informations: ecological information Other informations: use not remembered	4
*Scrophularia nodosa* L. (Scrophulariaceae) BCN3762	Setge	RI	Whole plant	Folk oral literature	1
*Secale cereale* L. (Poaceae) BCN27243	Secle, sègol	MO, RI	Fruit. Stem. Whole plant	Domestic: for help at home Ludic Magic and religious beliefs and practices	3
*Sechium edule* (Jacq.) Sw. (Cucurbitaceae) BC114504	Tayote (Spanish)	BI	Whole plant	Other informations: harvesting and/or selling	1
*Sedum dasyphyllum* L. (Crassulaceae) BCN24994	Arròs de paret	RI	Aerial part. Leaf. Whole plant	Ludic Ornamental: bouquet elaboration	3
*Sedum rupestre* L. (Crassulaceae) BCN29791	Mort-i-viu	AE	Aerial part	Magic and religious beliefs and practices	1
*Sedum sediforme* (Jacq.) Pau (Crassulaceae) BCN29792	Arròs, crespinell, mort-i-viu, pinet, raïm de galàpet, raïm de llop	AE, MO	Aerial part	Ludic Magic and religious beliefs and practices Ornamental: bouquet elaboration	4
*Sedum telephium* L. (Crassulaceae) BCN24995	Matafoc	RI	Whole plant	Ornamental: bouquet elaborations	1
*Sedum telephium* L. subsp. *maximum* (Crassulaceae) BCN32123	Bàlsam, bàlsam de fulla, bàlsam de jardí, fava de jardí, fava grassa	MO	Whole plant	Ornamental: gardening	2
*Sempervirum arachnoideum* L. (Crassulaceae) BCN26422	Vel de núvia	RI	Whole plant	Ornamental: gardening	1
*Sempervirum tectorum* L. (Crassulaceae) BCN24997	Apagafoc, consolva, herba de les cremades, matafoc, pinya de foc, sempervívum	MO, RI	Whole plant	Ornamental: bouquet elaboration Ornamental: gardening	4
*Senecio inaequidens* DC. (Asteraceae) BCN31309	Seneci del Cap	AE	Whole plant	Other informations: ecological information	2
*Sequoiadenderon giganteum* (Lindl.) J.Buchholz (Taxodiaceae) BCN27276	Sequoia	RI	Bark	Ludic	1
*Setaria viridis* (L.) Beauv. (Poaceae) BCN52592	Cua de mula, panissola, pèl de boc, xerreix, xerreix bord	MO	Aerial part	Ornamental: bouquet elaboration	1
*Sideritis* cf. *sventenii* (Kunkel) Mend.-Heu (Lamiaceae) BCN29957	_	AE	Aerial part	Ornamental: bouquet elaboration	1
*Silene vulgaris* (Moench) Garcke (Caryophyllaceae) BCN29948	Colís, colitx, colleja (Spanish), culivells, esclafidor, petapetó	AE, BI, MO	Aerial part. Flower. Leaf. Whole plant	Ludic Ornamental: bouquet elaboration Other informations: use not remembered	22
*Silene vulgaris* (Moench) Garcke subsp. *vulgaris* (Caryophyllaceae) BCN25001	Corretjola, esclafidor, pet	RI	Aerial part. Flower	Ludic	2
*Sinapis alba* L. (Brassicaceae) BCN50358	Mostassa	BI	Whole plant	Agrosilvopastoral management: tools for agricultural practices Repellent	2
*Smilax aspera* L. (Smilacaceae) BCN21151	Aríjol, aritja, sarsaparrilla	AE, BI	Aerial part. Root. Whole plant	Agrosilvopastoral management: for pig slaughter Domestic: for help at home Ornamental: bouquet elaboration Other informations: ecological information Other informations: use not remembered Textile: textile padding	10
*Smyrnium olusatrum* L. (Apiaceae) BNC95551	Aleixandre, aleixandri	BI	Aerial part. Stem	Artisanal: broom elaboration Ornamental: bouquet elaboration	3
*Solanum linnaeanum* Hepper & P.-M.L.Jaeger (Solanaceae) BCN2553	Metzinera, poma del desert	BI	Whole plant	Other informations: use not remembered	1
*Solanum lycopersicum* L. (Solanaceae) BCN29952	Domàtiga (fruit), domatiguera, tomata (fruit), tomatera, tomàtiga (fruit), tomatiguera,	AE, BI	Fruit. Leaf. Whole plant	Agrosilvopastoral management: agricultural/horticultural Domestic: for help at home Other informations: ecological information Other informations: harvesting and/or selling Repellent Smoking	9
*Solanum melongena* L. (Solanaceae) BCN25004	Albergínia (fruit), alberginiera	BI	Fruit. Leaf. Whole plant	Agrosilvopastoral management: agricultural/horticultural Magic and religious beliefs and practices Smoking	3
*Solanum tuberosum* L. (Solanaceae) BCN29797	Patata (tuber), patatera	AE, BI, MO	Leaf. Stem. Tuber. Whole plant	Agrosilvopastoral management: agricultural/horticultural Domestic: for help at home Magic and religious beliefs and practices Smoking	7
*Sonchus oleraceus* (L.) L. (Asteraceae) BCN29953	Allitsó, allitsó vertader, alitxó, aritsó, lletsó, lletxó, llitsó, llitxó	BI	Latex. Whole plant	Other informations: ecological information	2
*Sonchus tenerrimus* L. (Asteraceae) BCN29954	Aletxó, llitsó bord	BI	Leaf	Other informations: ecological information	1
*Sorbus aucuparia* L. (Rosaceae) BCN25009	Moixera	RI	Whole plant	Ornamental: gardening	1
*Sorbus domestica* L. (Rosaceae) BCN46827	Serva (fruit), server, servera	AE, BI, MO	Stem. Whole plant	Agrosilvopastoral management: tools for agricultural practices Other informations: ecological information Timber	7
*Sorghum bicolor* (L.) Moench (Poaceae) BCN31310	Melca	AE	Stem	Artisanal: broom elaboration	13
*Sorghum halepense* (L.) Pers. (Poaceae) BCN44695	Canyota	MO	Aerial part	Ornamental: bouquet elaboration	2
*Spartium junceum* L. (Fabaceae) BCN29956	Ginesta, ginesta de llei, ginesta vera, ginestera	AE, BI, MO, RI	Aerial part. Flower. Stem. Whole plant	Agrosilvopastoral management: tools for agricultural practices Artisanal: broom elaboration Artisanal: musical instrument elaboration Domestic: for help at home Magic and religious beliefs and practices Ornamental: bouquet elaboration Ornamental: gardening Other informations	27
*Spinacia oleracea* L. (Amaranthaceae) BCN46077	Espinac	BI	Leaf	Other informations: appreciations on plant or product properties	1
*Stachys byzantina* K.Koch (Lamiaceae) BCN126667	Bàlsam, bàlsam de tall, bàlsam pelut, herba peluda, orella de conill	MO	Whole plant	Ornamental: gardening	2
*Stipa tenacissima* L. (Poaceae) BCN46091	Clin, espart	AE, BI, MO	Aerial part. Stem. Whole plant	Agrosilvopastoral management: tools for agricultural practices Agrosilvopastoral management: for hunting Artisanal: basketry Artisanal: footwear elaboration Domestic: for help at home Magic and religious beliefs and practices Other informations: use not remembered Textile: textile padding Timber: boat manufacturing	11
*Syringa vulgaris* L. (Oleaceae) BCN29959	Lilà	AE, MO, RI	Aerial part. Flower. Whole plant	Folk oral literature Magic and religious beliefs and practices Ornamental: bouquet elaboration Ornamental: gardening	17
*Syzygium aromaticum* (L.) Merr. & L.M.Perry (Myrtaceae) BCN47279	Clau, clau d’olor	BI	Floral bud	Domestic: air freshener Repellent	2
*Tamarix canariensis* Willd. (Tamariaceae) BCN43378	Tamariu	AE	Whole plant	Folk oral literature	1
*Tanacetum corymbosum* (L.) Sch.Bip. (Asteraceae) BCN31918	_	MO	Whole plant	Other informations: use not remembered	1
*Tanacetum parthenium* (L.) Sch.Bip. (Asteraceae) BCN25014	Camamilla amargant, camamiglla borda, camamilla de Sòria, camamilla purgant, camamil · la de jardí, botonets	BI, MO, RI	Aerial part. Whole plant	Ornamental: bouquet elaboration Ornamental: gardening	3
*Tanacetum vulgare* L. (Asteraceae) BCN29803	Danarida, tanaceto (Spanish), tanarida	BI, RI	Aerial part. Whole plant	Agrosilvopastoral management: agricultural/horticultural Ornamental: bouquet elaboration Repellent	4
*Taraxacum officinale* Weber in Wiggers (Asteraceae) BCN25015	Angelets, dent de lleó, herba queixalera, lleones, llums, màstecs bords, paraigües, pixacà, pixaconills, pixallits, puputs, queixal de llop, xicoina	BI, MO, RI	Aerial part. Inflorescence. Fruit. Whole plant	Cosmetic Ludic Magic and religious beliefs and practices	8
*Taxus baccata* L. (Taxaceae) BCN25017	Teix, quiner	BI, MO, RI	Aril. Cortical parenchyma. Stem. Whole plant	Cosmetic Folk oral literature Ludic Magic and religious beliefs and practices Other informations: ecological information Timber: furniture manufacturing	6
*Teucrium capitatum* L. (Lamiaceae) BCN43441	Herba de Sant Ponç, mançanella	BI	Aerial part	Ornamental: bouquet elaboration Other informations: use not remembered	2
*Teucrium chamaedrys* L. (Lamiaceae) BC806359	Auladella, betera	BI	Whole plant	Other informations	1
*Teucrium chamaedrys* L. subsp. *pinnatifidum* (Sennen) Rech.f. (Lamiaceae) BCN126671	Brutònica	MO	Flower	Agrosilvopastoral management: tools for agricultural practices Other informations: ecological information	1
*Teucrium marum* L. subsp. *subspinosum* (Pourr. ex Willd.) O.Bolòs, Molin. & P.Monts. (Lamiaceae) BCN66404	Auladella, beteta, coixinet de monja, eixorba-rates, herba beteta, herba de Sant Ponç, poniol	BI	Whole plant	Agrosilvopastoral management: weeds Folk oral literature	2
*Teucrium polium* L. subsp. *capitatum* (L.) Arcang. (Lamiaceae) BCN103557	Auladella, beteta, coixinet de monja, eixorba-rates, herba beteta, herba de Sant Ponç, poniol	BI	Aerial part	Ornamental: bouquet elaboration	1
*Teucrium polium* L. subsp. *polium* (Lamiaceae) BCN32116	Timó, timolet, timonet	MO	Flower	Agrosilvopastoral management: tools for agricultural practices	1
*Thapsia villosa* L. subsp. *villosa* (Apiaceae) BCN39998	Julivert de cavall	AE	Whole plant	Other informations: ecological information	1
*Thymbra capitata* (L.) Cav. (Lamiaceae) BCN72716	Frígola	BI	Leaf	Domestic: for help at home	1
*Thymus vulgaris* L. (Lamiaceae) BCN29961	Farigola, frigola, tem, tomillo (Spanish)	AE, BI, RI	Aerial part. Flower	Cosmetic Domestic: air freshener Magic and religious beliefs and practices Other informations: excipient or adjuvant in medicinal or food preparations	8
*Thymus vulgaris* L. subsp. *vulgaris* (Lamiaceae) BCN44665	Farigola	MO	Aerial part	Agrosilvopastoral management: agricultural/horticultural Folk oral literature	6
*Tilia cordata* Mill. (Malvaceae) BCN26784	Til · lo	BI	Bract with flower	Other informations: harvesting and/or selling	2
*Tilia platyphyllos* Scop. (Malvaceae) BCN25024	Flor de tei, tell, til · la	AE, RI	Inflorescence. Whole plant	Ornamental: gardening Other informations: harvesting and/or selling	3
*Tilia* sp. (Malvaceae)	Til · la	BI	Whole plant	Other informations: use not remembered	1
*Tillandsia* sp. (Bromeliaceae)	Clavell de l’aire	BI	Whole plant	Ornamental: gardening	1
*Tradescantia albiflora* Kunth. (Commelinaceae) BCN49624	_	MO	Whole plant	Ornamental: gardening	1
*Tribulus terrestris* L. (Zygophyllaceae) BCN45017	Picatalons	BI	Whole plant	Agrosilvopastoral management: weeds	1
*Trichocereus pachanoi* Britton & Rose (Cactaceae) BCN129086	Dompedro (Spanish), sampedro (Spanish)	BI	Aerial part. Whole plant	Magic and religious beliefs and practices Ornamental: gardening Other informations: appreciations on plant or product properties	4
*Trifolium* sp. (Fabaceae)	Trèvol	BI	Aerial part. Whole plant	Agrosilvopastoral management: tools for agricultural practices Fuel obtaining: charcoal Other informations: ecological information	4
*Triticum aestivum* L. (Poaceae) BCN29963	Blat, segó	AE, BI, MO, RI	Aerial part. Bran. Not reported. Fruit. Spike. Stem. Whole plant	Agrosilvopastoral management: agricultural/horticultural Agrosilvopastoral management: for fishing Artisanal: footwear elaboration Cosmetic Domestic: for help at home Folk oral literature Magic and religious beliefs and practices Ornamental: bouquet elaboration Other informations: ecological information Other informations: collections and/or selling Other informations: use not rememberd	24
*Trollius europaeus* L. (Ranunculaceae) BCN27267	Cloclou, flor de Sant Pere, rovell d’ou	RI	Aerial part	Ornamental: bouquet elaboration	2
*Tropaeolum majus* L. (Tropaeolaceae) BCN46429	Canari, caputxina	BI, MO	Whole plant	Agrosilvopastoral management: tools for agricultural practices Ornamental: gardening	2
*Tulbaghia* sp. (Amaryllidaceae)	Tulbàgia	BI	Whole plant	Agrosilvopastoral management: tools for agricultural practices Repellent	2
*Tulipa* sp. (Liliaceae)	Tulipán (Spanish)	BI	Whole plant	Ornamental: gardening	1
*Typha angustifolia* L. (Typhaceae) BCN23969	Boga, bova	BI, MO	Aerial part. Leaf. Stem	Folk oral literature Ornamental: bouquet elaboration Textile: fibre or cloth elaboration	12
*Typha angustifolia* L. subsp. *australis* (Schumach.) Kronf. (Typhaceae) BCN51627	Bova	BI	Stem	Artisanal: basketry Other informations: ecological information Textile	3
*Typha latifolia* L. (Typhaceae) BCN31314	Balca, boga, bova	AE, BI, MO	Aerial part. Flower. Leaf. Stem	Agrosilvopastoral management: tools for agricultural practices Artisanal: basketry Folk oral literature Ludic Ornamental: bouquet elaboration Other informations: ecological information Textile: fibre or cloth elaboration Timber: furniture manufacturing	23
*Ulex parviflorus* Pourr. subsp. *parviflorus* (Fabaceae) BCN30011	Argelac, argentina	AE	Stem. Whole plant	Fuel obtaining: firewood Other informations: ecological information	2
*Ulmus minor* Mill. (Ulmaceae) BCN29813	Om	AE, BI, MO, RI	Bark. Stem	Agrosilvopastoral management: agricultural/horticultural Artisanal: cane elaboration Artisanal: cookware elaboration Folk oral literature Textile: fibre or cloth elaboration Timber Timber: boat manufacturing Timber: wheeled vehicles manufacturing Timber: furniture manufacturing Timber: construction materials production	20
*Urginea maritima* (L.) Baker (Asparagaceae) BCN58049	Ceba marina, ceba marinera	BI	Bulb. Leaf. Whole plant	Agrosilvopastoral management: tools for agricultural practices Ornamental: gardening Other informations: ecological information Repellent	16
*Urtica dioica* L. (Urticaceae) BCN25030	Ortiga	MO, RI	Aerial part. Whole plant	Folk oral literature Magic and religious beliefs and practices	2
*Urtica membranacea* Poir. ex Savigny (Urticaceae) BCN95578	Ortiga	BI	Aerial part. Whole plant	Agrosilvopastoral management: tools for agricultural practices Cosmetic Other informations	5
*Urtica* sp. (Urticaceae)	Ortiga	BI	Aerial part. Leaf. Whole plant	Agrosilvopastoral management: tools for agricultural practices Folk oral literature Magic and religious beliefs and practices Other informations: undesirable actions Repellent	6
*Urtica urens* L. (Urticaceae) BCN29966	Estrígol, ortiga	AE, BI	Aerial part. Leaf. Not reported. Stem. Whole plant	Agrosilvopastoral management: tools for agricultural practices Cosmetic Other informations: undesirable actions Repellent	7
*Valeriana officinalis* L. (Caprifoliaceae) BCN29816	Valedriana, valeriana	AE, MO, RI	Root	Folk oral literature	6
*Verbascum pulverulentum* Vill. (Scrophulariaceae) BCN25035	Herba ploranera, torpa	RI	Aerial part	Agrosilvopastoral management: weeds Magic and religious beliefs and practices	2
*Verbascum sinuatum* L. (Scrophulariaceae) BCN29967	Trepó, tripó	AE, BI	Root. Stem. Whole plant	Artisanal: broom elaboration Folk oral literature Magic and religious beliefs and practices	4
*Verbascum* sp. (Scrophulariaceae)	Croca, cua de guilla, trepó	BI, RI,	Aerial part. Leaf. Not reported. Whole plant	Domestic: for help at home Magic and religious beliefs and practices Other informations: ecological information	7
*Verbascum thapsus* L. (Scrophulariaceae) BCN63299	Porpra, trepó	MO	Flower	Cosmetic	1
*Verbascum thapsus* L. subsp. *montanum* (Schard.) Bon (Scrophulariaceae) BCN39651	Candeler, trepó	BI	Flower. Leaf	Folk oral literature Other informations: ecological information	2
*Viburnum lantana* L. (Adoxaceae) BCN27281	Cartellatge, tortellatge	AE, MO, RI	Aerial part. Stem	Agrosilvopastoral management: tools for agricultural practices Artisanal: cane elaboration Magic and religious beliefs and practices	8
*Viburnum opulus* L. (Adoxaceae) BCN126683	Bola de neu, mató, pompa	MO, RI	Aerial part. Whole plant	Ornamental: bouquet elaboration Ornamental: gardening	2
*Viburnum opulus* L. var. *roseum* Aiton (Adoxaceae) BCN27254	Mató	RI	Aerial part	Magic and religious beliefs and practices	1
*Viburnum tinus* L. subsp. *tinus* (Adoxaceae) BCN126681	Marfull	MO	Aerial part. Whole plant	Agrosilvopastoral management: tools for agricultural practices Ornamental: gardening	3
*Vicia cracca* L. subsp. *gerardii* (W.D.J. Koch) Briq. (Fabaceae) BCN126685	Llegum, veça	MO	Aerial part	Magic and religious beliefs and practices	1
*Vicia faba* L. (Fabaceae) BCN49339	Fava (fruit/seed), favera, favó (seed), llegum	BI, RI	Fruit. Seed. Whole plant	Agrosilvopastoral management: agricultural/horticultural Agrosilvopastoral management: honey obtaining Folk oral literature Magic and religious beliefs and practices Other informations: harvesting and/or selling	13
*Vicia sativa* L. (Fabaceae) BCN95569	Veça	BI	Young shoot	Magic and religious beliefs and practices Ornamental: bouquet elaboration	2
*Vicia sativa* L. subsp. *sativa* (Fabaceae) BCN-E-93	Veça	MO	Whole plant	Ornamental: gardening	1
*Vicia villosa* Roth (Fabaceae) BCF46443	Veça	MO	Whole plant	Magic and religious beliefs and practices	1
*Vinca difformis* Pourr. (Apocynaceae) BCN52597	Blincaperblinca, proenga, pruenga, vinca, vincapervinca	BI, MO, RI	Aerial part. Whole plant	Folk oral literature Ornamental: bouquet elaboration Other informations: ecological information	5
*Vinca major* L. (Apocynaceae) BCN25039	Flor de Pasqua, vinca, vincapervinca	AE, RI	Aerial part. Whole plant	Ornamental: bouquet elaboration Ornamental: gardening	4
*Viola alba* Besser (Violaceae) BCN27286	Violetes	RI	Flower	Ornamental: bouquet elaboration	1
*Viola alba* Besser. subsp. *dehnhardtii* (Ten.) W.Becker (Violaceae) BCN31316	Viola, viola petita	AE	Aerial part	Ornamental: bouquet elaboration	1
*Viscum album* L. (Santalaceae) BCN46085	Gui (French), muérdago (Spanish), ramillo de la suerte (Spanish), vesc	AE, MO	Aerial part. Resin. Whole plant	Agrosilvopastoral management: for hunting Magic and religious beliefs and practices Ornamental: bouquet elaboration	7
*Vitex agnus-castus* L. (Lamiaceae) BCN29820	Alís d’olor, aloc, barda, ximbla	AE, BI	Flower. Latex. Stem	Agrosilvopastoral management: tools for agricultural practices Ludic Magic and religious beliefs and practices Other informations: use not remembered Repellent	5
*Vitis vinifera* L. (Vitacaeae) BCN29972	Cep, pansa (dried fruit), tòria (branch), parra, raïm (fruit), raïmera, rem (fruit), vi (elaborated product), vinagre (elaborated product)	AE, BI, RI	Fruit juice. Leaf. Fruit peduncle Stem. Whole plant.	Agrosilvopastoral management: agricultural/horticultural Agrosilvopastoral management: for pig slaughter Cosmetic Domestic: for help at home Folk oral literature Fuel obtaining: firewood Magic and religious beliefs and practices Ornamental: gardening Other informations: excipient or adjuvant in medicinal or food preparations Repellent Smoking	35
*Wisteria sinensis* (Sims) Sweet (Fabaceae) BCN30014	Glicina	AE, BI	Whole plant	Ornamental: gardening	2
*Zantedeschia aethiopica* (L.) Spreng. (Araceae) BCN104895	Lliri	BI	Aerial part. Whole plant	Ornamental: bouquet elaboration Ornamental: gardening	3
*Zea mays* L. (Poaceae) BCN29830	Blat de les Índies, blat de moro, cabellet (style and stigma), maís	AE, BI, MO, RI	Bract, Epicarp, Fruit. Infructescence. Not reported. Fruit. Stem. Spike. Styles and stigmas. Whole plant. Rachis	Agrosilvopastoral management: agricultural/horticultural Fuel obtaining: firewood Ludic Magic and religious beliefs and practices Ornamental: bouquet elaboration Smoking Textile: dyer Textile: fibre or cloth elaboration Textile: textile padding Other informations	27
*Zinnia elegans* L. (Asteraceae) BCN69604	Rascamoño (Spanish), rosa mística	BI	Inflorescence. Whole plant	Agrosilvopastoral management: tools for agricultural practices Ornamental: bouquet elaboration Ornamental: gardening	3
*Ziziphus jujuba* L. (Rhamnaceae) BCN29822	Gíjol (fruit), ginjoler	AE, BI, MO	Aerial part. Fruit. Stem. Whole plant	Artisanal: musical instrument elaboration Magic and religious beliefs and practices Other informations: ecological information Timber: boat manufacturing	11

### Statistical methods

All calculations were carried out using Excel (Microsoft Excel 2007) and the program XLSTAT (v. 2007.5, Addinsoft SARL) was used to carry out the Chi-square test in order to check the statistical differences in the use categories among the four studied territories.

## Results and discussion

### General data

The 769 informants reported 401 genera (34 of the taxa reported have only been determined to the generic level), 552 species, 81 subspecies and four varieties, belonging to 122 families. The taxa collected, their folk names and uses, the territories where they are recorded, the parts of plants employed and their use frequency are presented in Table 1, placed as an appendix. The number of taxa with such information is high. Just to compare with the same geographical areas, 334 species are reported in a paper on medicinal plants (AE, [30]), which is representative of other territories, a figure higher than in other areas and not simply additive, since a high number of taxa are repeated in different areas, and 97 species were recorded in a work on ethnoveterinary in four out of the five territories here considered [31]. This supports the argument to consider the non-food and non-medicinal folk plant uses as not secondary or residual at all. The number of use reports (hereinafter UR) confirms this, being also elevated (4137 in total, cf. Table 2 for their distribution in the geographical areas and Table 3 for its repartition for use categories).

**Table 2:** Use reports in the studied areas

Area	Use reports	Use reports/inhabitant	Use reports/informant
Catalonia (CA)^a^	2335	0.0086	4.58
Alt Empordà (AE)	1014	0.0072	5.70
Montseny (MO)	942	0.0090	5.48
Ripollès (RI)	379	0.0147	2.37
Balearic Islands (BI)^a^	1802	0.0021	6.96
Formentera (FO)	87	0.0075	3.63
Mallorca (MA)	1715	0.0020	7.30
TOTAL	4137	0.0036	5.38

^a^Catalonia includes Alt Empordà, Montseny and Ripollès and Balearic Islands include Formentera and Mallorca

**Table 3:** Use categories considered and number of reports of each one in the territories studied

Use categories	AE	BI	MO	RI
Agrosilvopastoral management	135	284	159	24
Artisanal	250	157	96	31
Cosmetic	26	78	9	12
Domestic	77	113	63	23
Folk oral literature	16	106	64	10
Fuel obtaining	16	58	27	9
Ludic	40	64	117	26
Magic and religious beliefs and practices	128	116	105	72
Ornamental	135	193	201	117
Other informations	79	383	13	33
Repellents	20	95	3	11
Smoking plant	24	18	19	3
Textile	25	58	1	4
Timber	43	79	65	4
TOTAL	1014	1802	942	379

*AE* Alt Empordà, *BI* Balearic Islands (comprising here Formentera and Mallorca), *MO* Montseny, *RI* Ripollès

To name the specific and infraspecific taxa the informants provided 1303 Catalan popular names, including whole plants and parts of plant, especially fruits and seeds, and elaborated products. This figure would be the equivalent to a mean of more than two names per plant, and roughly approaches the 5 % of the ca. 35,000 Catalan plant names (of folk and other provenances) recently collected [32], the quantity and diversity of names accounting too for the vitality of the knowledge of plants with these other uses. Additionally, the informants indicated 37 Spanish, four French and one Arabic names to refer to the plants reported.

### Use categories

Table 3 presents the 14 categories (which are equally present for all territories in our database to avoid comparison) of non-food and non-medicinal uses in which we have classified all data collected. Some examples and comments on specific uses are provided in this and in other epigraphs of this paper (e.g., most reported taxa or persistence of uses), and the detail of all uses collected can be found in Table 1.

The category “other informations” is a miscellaneous one to hold sparse knowledge not attributable to the established categories. This big group of information comprises 509 use reports from a total 4137, meaning ca. 12 % of all reports, but includes a very numerous and diverse cases, for which we have established the following subgrouping: ecological information, undesirable actions, purchased in commerce, harvesting and/or selling, excipient or adjuvant in medicinal or food preparations, product appreciations. We also attributed the category of other information to the cases in which the informants declared that they knew that a plant was useful, but they did not remember for what (use not remembered); we maintain this category (related, as the whole paper, to non-food and non-medicinal uses) as an evocation of knowledge that used to be solid, but has declined and is almost forgotten now.

Some big categories comprise several activities. Agrosilvopastoral: agricultural/horticultural, for pig slaughter, honey obtaining, for fishing, for hunting, forestry tools for agricultural practices, weeds. Artisanal: elaboration of canes, shoes, brooms, kitchen implements, musical instruments, baskets and similar objects, fibres, smoking paper, tobacco pipes and other instruments, as well as hide tanning. Domestic: household help, air fresheners, paint elaboration, help in sewing. Textile: fibre or cloth elaboration, textile padding, dyer. Timber: elaboration of furniture, boats and wheeled vehicles, obtention of building material.

With this, and irrespective of our grouping, made for the sake of concision, thus, the number of subcategories (including categories with no subcategories) would reach 46, showing a big coverage of different fields. The most common large use categories, in terms of number of use reports, are ornamental (646 use reports), agrosilvopastoral management (600), artisanal (534), and magic and religious beliefs and practices (421). For statistical purposes we used the main or big categories, since for many of the subcategories there are not enough use reports to allow a statistical treatment.

**Fig. 2:**
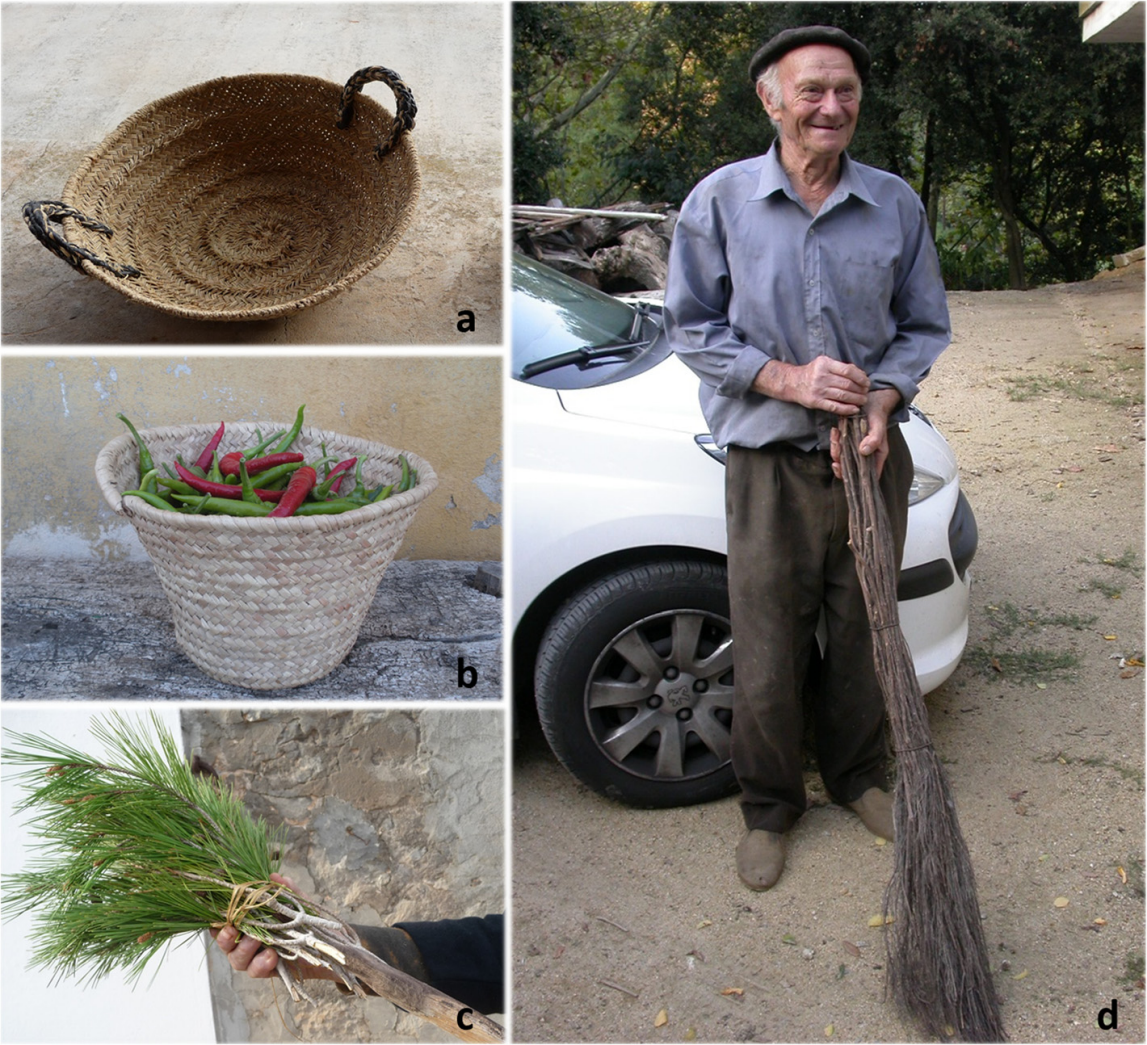
Examples of basketry and broom elaboration in the territories considered: a. Formentera, *Lygeum spartum*; b. Mallorca, *Chamaerops humilis*; c. Formentera, *Juniperus phoenicea*, *Pinus halepensis*; d. Montseny, *Erica scoparia*

The first two of these categories have direct links with the rural society, which has been prevalent in all the studied territories until recent times and is still important, irrespective of the intervening socioeconomic changes. Plants used in agricultural and livestock raising tasks have been and many of them continue to be utilised (see the subheading on persistence of uses). Many local handicrafts and objects for domestic uses were and are elaborated. Many kinds of baskets (Fig. 2), spoons and other kitchen implements, often made with *Buxus sempervirens* L. wood, or broom (Fig. 2; made with species such as *Buxus sempervirens*, *Erica scoparia* L., *Mantisalca salmantica* (L.) Briq. et Cavill. and *Sarothamnus scoparius* (L.) W.D.J.Koch -two of them with the specific epithet alluding to this object, suggesting the antiquity and relevance of this tradition-) were mentioned. The uses comprised in these two categories are also among the commonest in other European areas [11, 13, 14]. Moreover, handicraft elaboration has been reported as one of the most relevant uses in distant areas as well, e.g., [33]. Indeed, some uses belonging to other categories are also linked with rural activities. This is the case, among others, of using *Datura stramonium* L. as a repellent for moles, which can damage plant cultures.

**Fig. 3:**
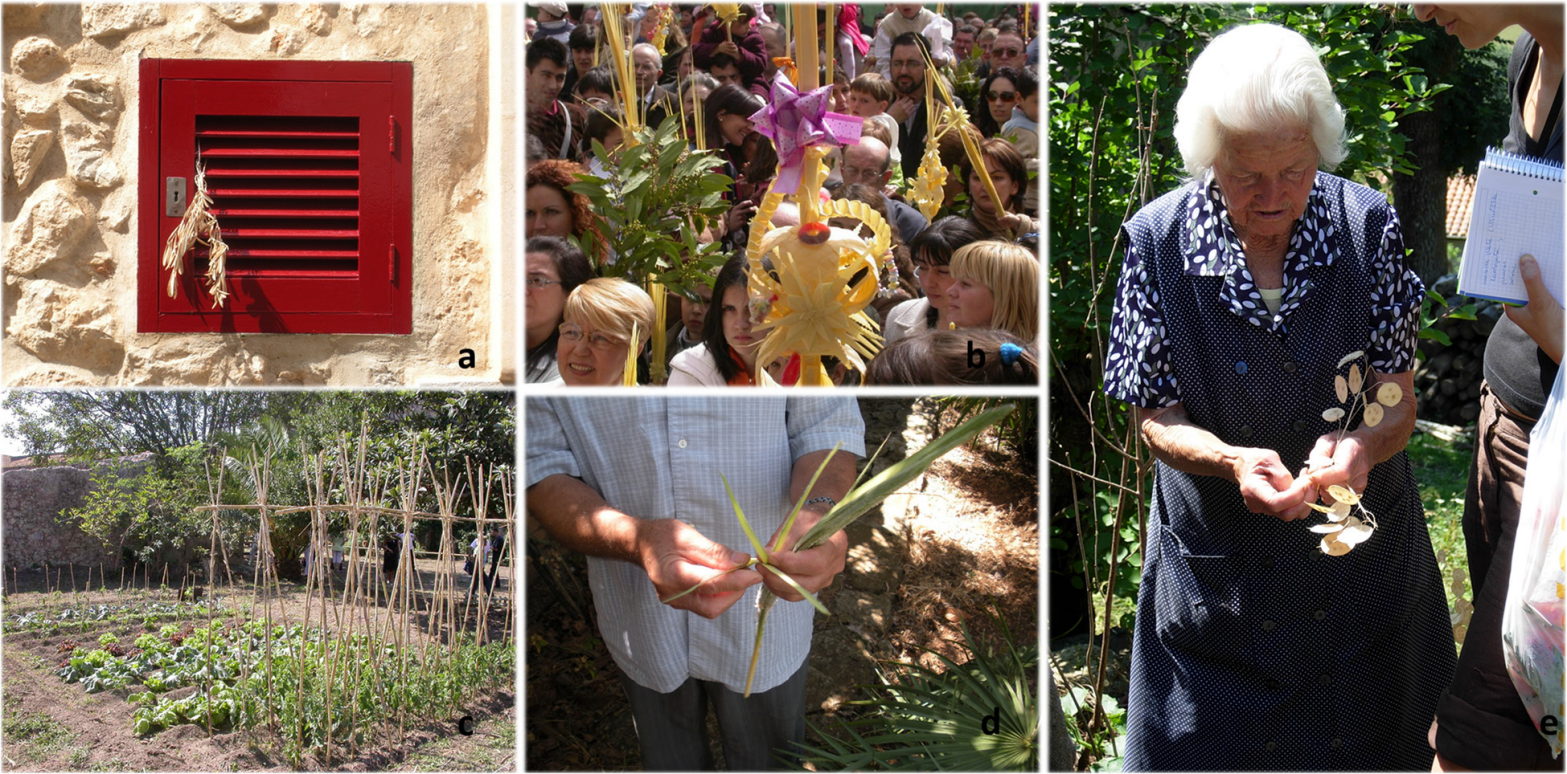
Examples of artisanal, domestic and magicoreligous uses in the territories considered: a. Mallorca, *Olea europaea*; b. Alt Empordà, *Laurus nobilis, Phoenix dactylifera*; c. Alt Empordà, *Arundo donax*; d. Mallorca, *Chamaerops humilis*; e. Ripollès, *Lunaria annua*

The third group of uses is very common as well, since all human groups have influences of religious and/or magic beliefs. Protective plants (see the subheading on persistence of uses), or ritual ones (e.g., *Laurus nobilis* L., *Olea europaea* L. and *Phoenix dactylifera* L., which are blessed in Christian Easter feasts, Fig. 3) exemplify this group of uses.

**Fig. 4:**
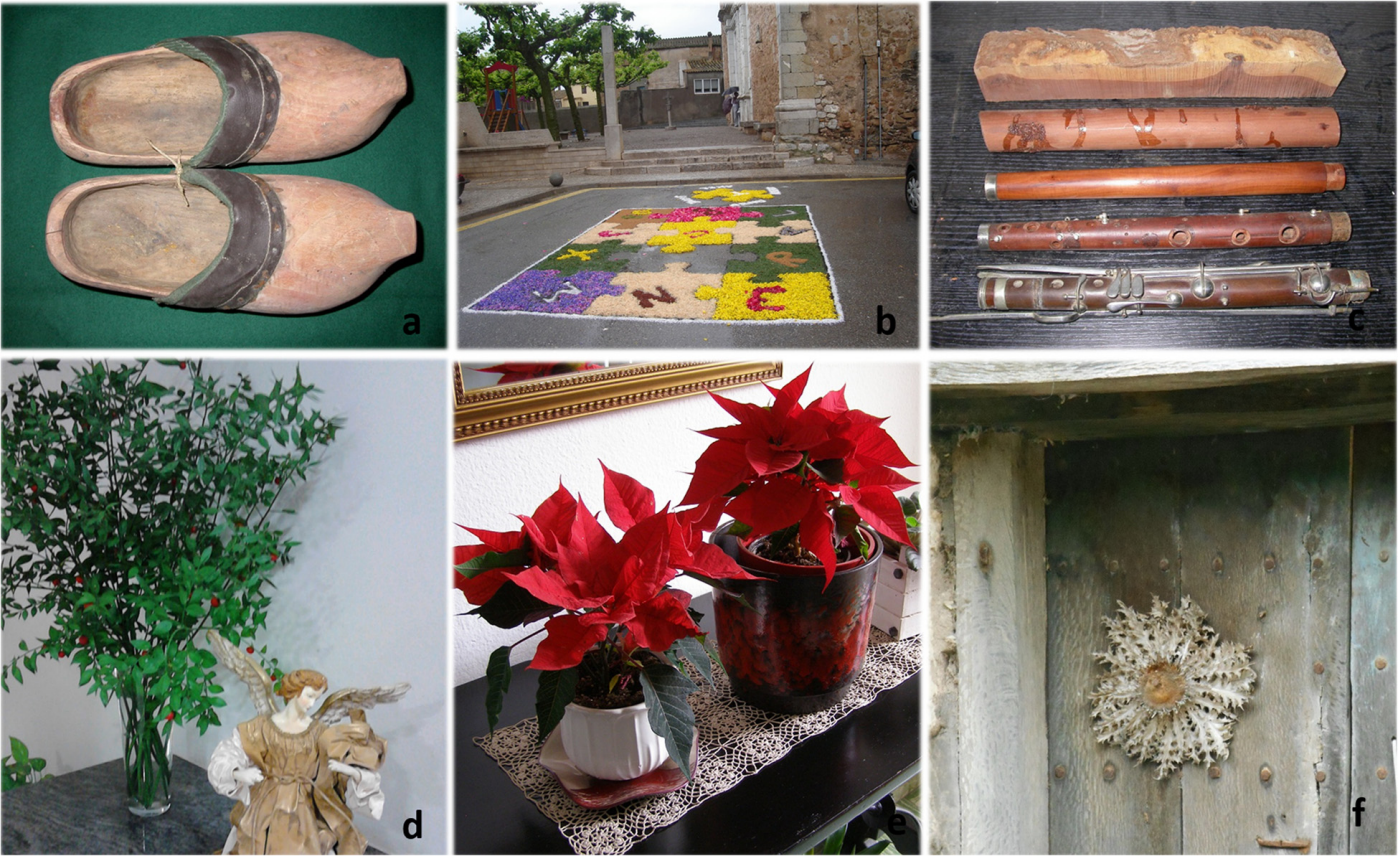
Examples of artisanal, domestic and magicoreligous uses in the territories considered: a. Alt Empordà, *Pinus pinea*; b. Alt Empordà, several species, among which *Dianthus caryophyllus*, *Malva sylvestris*, *Rosa* sp., *Spartium junceum* and *Phoenix dactylifera*; c. Alt Empordà, *Ziziphus jujuba*; d. Montseny, *Ruscus aculeatus*; e. Montseny, *Poinsettia pulcherrima*; f. Ripollès, *Carlina acanthifolia* subsp. *cynara*

Finally, the category of ornamental plants is also universal, both in rural and urban areas, both with living or cut plants, and both for indoor and outdoor spaces. This group of uses involves classical, large-scale cultivated plants (such as *Rosa* sp. cultivars), but a large number of wild plants as well, such as, among many others (see Table 1), *Lunaria annua* L. (Fig. 3), with the aerial parts with fruiting septa of which bouquets are prepared. Many ornamental plants also have other uses; amongst them, medicinal is not rare, and in some cases the medicinal application used to be important and has decreased, but the ornamental use persists (such as, for example, in *Lilium candidum* L.). Decorative uses are very dynamic. The ancient traditions in this field coexist with recently adopted ones, such as using *Poinsettia pulcherrima* (Willd.) Graham toghether with the classical *Ruscus aculeatus* L. for home ornamentation at Christmas (Fig. 4).

### Most reported taxa

The five most reported families in the whole studied area are Poaceae (301 UR; 7.28 %), Lamiaceae (293 UR; 7.08 %), Asteraceae (290 UR; 7.01 %), Rosaceae (186 UR; 4.50 %) and Oleaceae (181 UR; 4.38 %). All these families, except the last one, are big in terms of number of taxa and are importantly represented worldwide and, in particular, in the Mediterranean region, to which the territories considered belong. This would agree with the assessment that plant taxa common or well-represented in one area are more accessible to its inhabitants and, so, most likely used there [34]. Three of these families, Asteraceae, Lamiaceae and Rosaceae, are almost always dominant in ethnobotanical works conducted in the Catalan cultural area and in other western Mediterranean countries (see, for instance, [35] for a Catalan territory and [36, 37] for non-Catalan areas, and references contained in these papers) even if those works are focused on food and medicinal plants and the present one on other uses. The coincidence is also important (four out of the five main families of the present paper) even with work conducted in several eastern Mediterranean areas [14]. In contrast, the family Oleaceae is not very numerous in taxa, but it reached a very high number of UR mostly due to the relevance and versatility of uses of *Olea europaea* (including, but not only, olive oil, utilised for many different purposes) in all Mediterranean territories. The family Poaceae is not included among the most reported in the above-quoted ethnobotanical works on food and medicinal plants, but is, again, a very large one and with some prominent plants in the kind of uses here studied. *Arundo donax* L. is particularly significant in this sense. This allochthonous plant, acclimated from ancient times in the areas studied, has many uses, mostly linked to agricultural practices (Fig. 3), in three of the territories (AE in Catalonia and both from BI), which account for many UR.

The five most cited species in the different regions studied are presented in Table 4. Again, *Olea europaea* is present in the top plants list, being the most reported one in the Balearic areas considered and in one of the Catalonian territories (altogether, and logically, in the most strict Mediterranean areas among those studied) for the reasons stated in the last paragraph. The coincidence of *Buxus sempervirens* as highly quoted in the three Catalonian territories responds, apart from its popularity due to the high quality of its wood, to the existence of Eurosiberian zones in these continental areas, and to its practical or total absence from Balearic ones. Conversely, *Chamaerops humilis* (Figs. 2 and 3), much more present in the Balearic areas considered, and scarce or inexistent in the Catalonian ones, is, not surprisingly, highly reported in the former. Most quoted taxa are quite different from those leading the lists of top plants for medicinal or food uses in the areas considered ([28, 38] and references therein), with the exception of *Olea europaea*, which also plays a relevant role in the mentioned fields.

**Table 4:** Data on use reports of the most quoted plants in the territories studied

Territory/Plant species	Number of use reports in each territory	Percentage of use reports in each territory
Alt Empordà		
*Olea europaea* L. (including both subsp. *europaea* and subsp. *sylvestris* (Mill.) Hegi)	44	4.34
*Arundo donax* L.	40	3.94
*Erica arborea* L.	39	3.85
*Celtis australis* L.	29	2.86
*Buxus sempervirens* L.	28	2.76
Ripollès		
*Buxus sempervirens*	28	7.39
*Carlina acanthifolia* (Pourr. ex DC.) Rouy	14	3.69
* Sarothamnus scoparius* L.	13	3.43
* Syringa vulgaris* L.	12	3.17
* Pinus sylvestris* L.	9	2.37
Montseny		
* Buxus sempervirens*	69	7.11
* Erica scoparia* L.	30	3.18
* Helichrysum stoechas* (L.) Moench.	29	3.08
* Pteridium aquilinum* (L.) Kuhn	24	2.55
*Quercus ilex* L. and *Scirpus holoschoenus* L., *ex aequo*	20	2.12
Balearic Islands (Mallorca and Formentera)		
*Olea europaea* L. (including both subsp. *europaea* and subsp. *sylvestris*)	80	4.44
* Arundo donax*	57	3.16
* Ocimum basilicum* L.	47	2.61
* Chamaerops humilis* L.	46	2.55
*Pinus halepensis* Mill. and *Prunus dulcis* (Mill.) D.A.Webb., *ex aequo*	41	2.28

### Parts of plants

The use categories object of the present paper being very diverse, almost all plant parts (and the whole plants as well) are used.

Stem (1156 UR; 27.94 %), aerial part -constituted either by all the aboveground part of the plant or by leaves and flowers together, with the stem or branch portions sustaining them- (785 UR; 18.98 %), flower (including inflorescence and flower, 337 UR; 8.15 %) and leaf (295 UR; 7.13 %) have been the most reported plant parts. Frequently, the whole plant is also used (869 UR; 21.01 %). Sometimes, the aerial part and the whole plant have constituted indistinguishable categories. The high number of use reports attributed to the stem is due to large and diverse kinds of artisanal uses, wood products and fuel.

### Comparison between the territories studied

As stated when addressing the most recorded taxa, the territories studied share a language and culture, but differ in ecogeographical characteristics. For these comparative purposes we have put together both Balearic territories, since Formentera’s dataset is not big enough to be taken into consideration.

Chi-square test showed there was an association between use categories and territories (χ^2^ = 846.306, d.f. = 39, *P* < 0.0001). Particularly, the presence of continental and insular areas leads us to suppose some differences in ethnoflora applications, possibly due to differences both in flora and in traditions. Indeed, there is also a significant difference in the use categories between continental and insular territories (χ^2^ = 485.037, d.f. = 13, *P* < 0.0001), confirming this geographical effect.

**Fig. 5:**
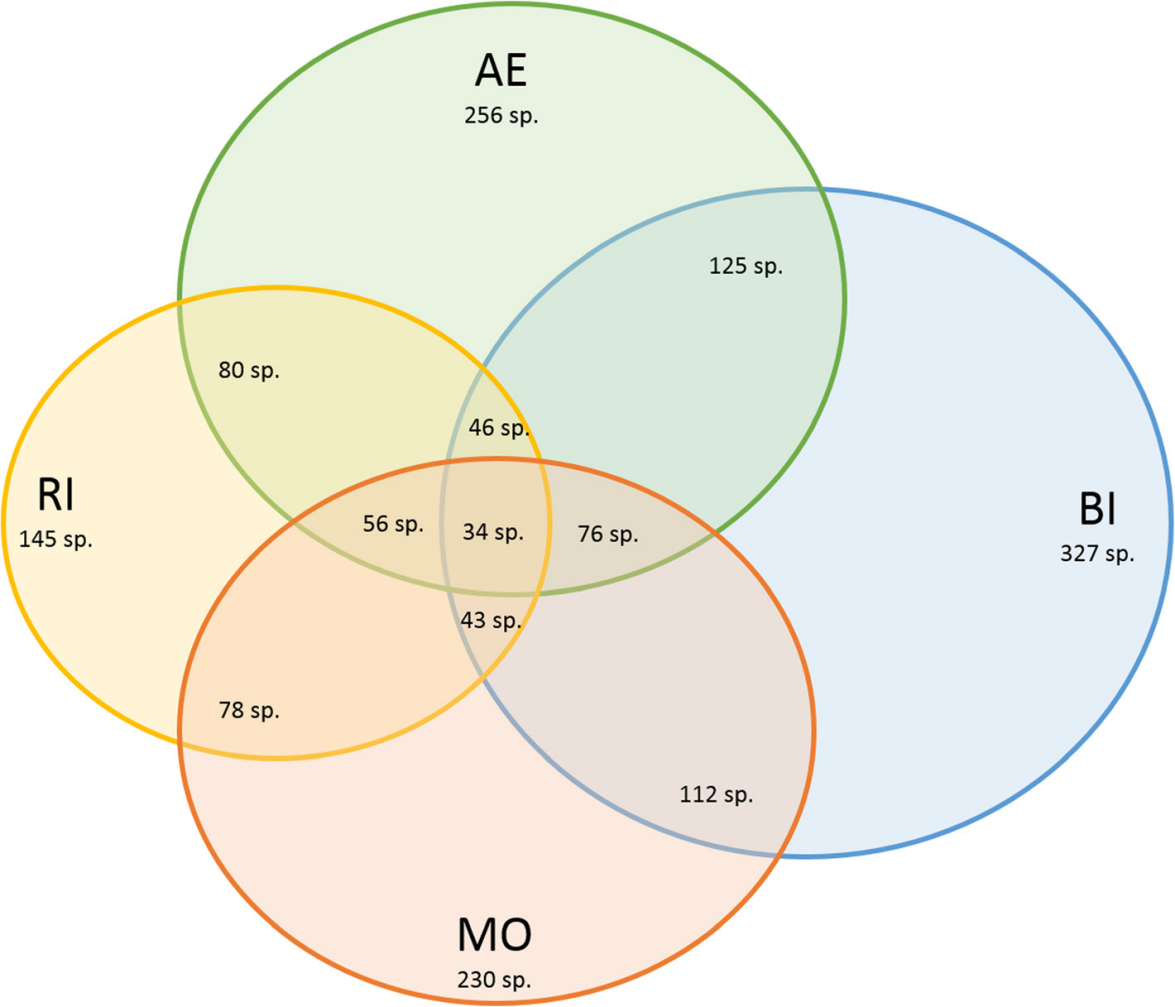
Venn’s diagram showing the number of uses in each territory considered and the coincidences among them. AE: Alt Empordà; BI: Balearic Isands; Mo: Montseny; RI: Ripollès

We present in Fig. 5 the degree of coincidence in plants used in the five regions considered. Whereas the number of coincident species among three territories ranges from 43 (Balearic Islands, Montseny and Ripollès) to 76 (Alt Empordà, Balearic Islands and Montseny), this decreases to 34 when we analyse the intersection of the four areas included in this study. In any case, we believe that the amount of plants used for non-medicinal and non-food aims in all areas is rather high. These plants with a larger geographical reach are usually cosmopolitan species used in daily life in the territories of Catalan culture, such as *Fagus sylvatica* L., *Laurus nobilis*, *Malva sylvestris* L., *Olea europaea*, *Papaver rhoeas* L., *Ruscus aculeatus*, and *Spartium junceum* L. (Table 1).

### Reliability of uses recorded

As already stated, the uses here reported are divided in a large number of categories. Irrespective of this dispersion, which is logical, since we consider here all traditional plant uses apart from food and medicinal ones, we think it is necessary to evaluate the degree of reliability of the information provided by the interviewees on these uses. A first, simple approach to this is considering the frequency of uses (see Table 1 for plants and Table 3 for use categories). According to several authors [33, 39], a use could be considered reliable if it is quoted by at least three independent informants. In most cases both the plants and the uses have been quoted by more (usually many more) than three informants.

A finer approach is provided by the informant consensus factor (F_IC_; [40]), defined as the number of UR minus the number of taxa divided by the number on UR minus 1. This index reflects the reliability of ethnobotanical information considering the coincidence of the informants in the same uses to be attributed to the same plants. The closer F_IC_ is to its maximum value, 1, the more consistent -thus, the more reliable- are the folk plant uses considered in the area studied. The general F_IC_ value in this study is 0.87 and those for the two big geographical areas concerned (Catalonia and Balearic Islands) are coincidental, 0.82. To our knowledge, this is the first, or one of the very rare times, that F_IC_ is calculated for uses other than medicinal and food ones. The absolute values can be considered rather high. Comparatively, they are higher than those calculated for medicinal uses in two Mexican and one Indian areas (ranging from 0.75 to 0.79; [41–43]). This means that other uses in the territories here studied are slightly more coincidental than medicinal uses in places where folk use of medicinal plants is highly practiced and considered, suggesting a high consistency and reliability of the information collected. The F_IC_ values here presented are very close (although in a few cases slightly lower) to those obtained for food and medicinal plant uses in the areas object of study ([31, 35], and references therein). This testifies for the robustness of the dataset collected, confirming the relevance of these other uses of plants for the human populations practicing them.

### Persistence and consideration of non-food and non-medicinal folk plant uses

If indices such as F_IC_ permit a quantitative assessment of the non-food and non-medicinal popular plant uses, the interviews performed with a big number of informants in the five studied areas allow us to qualitatively evaluate their current situation as well. Our aprioristic idea was that, in an industrialised society, these uses, most of them not directly linked to wellness and hardly related to rural life, would be residual and not much considered, at least in part due to the processes of acculturation and erosion of traditional knowledge. Nevertheless, once considered in detail the definitely high amount of information collected, we believe that these uses are (at least still) not so marginal. We contribute here some reflections on this subject with different possibilities for the uses considered, illustrated with a few examples, and more similar conclusions may be deduced by considering the information of Table 1.

Undoubtedly, some of these uses have declined, in some cases nearly or completely until extinction. We can note in this category some artisanal works and most traditions linked to religious and/or magic beliefs. For instance galoshes (Fig. 4), for which the wood of several trees was employed, are no longer in use in agropastoral tasks (so, they are no longer fabricated), but the informants who used to employ them and those who used to elaborate them (one of our informants was one of the last artisans who elaborated galoshes in Catalonia, until 1980) precisely remember the details of their use and had often brought into the house some pieces as decorative elements with an added value. In the Catalan society, the same degree as in the Netherlands -where galoshes have become an identitary element, with, additionally, a big implication in merchandising and trade- has not been reached, but nowadays the few people who know how to fabricate them are often required in popular feasts to show off their former trade. Indeed, courses or workshops on these and other subjects related with traditional plant uses (e.g., dyeing) are frequently organised. Similarly, carpets elaborated with vegetal elements are no longer used in religious processions (simply because these processions do not exist currently), but in some localities in two of the areas studied (Alt Empordà, Montseny) the carpets continue to be prepared every year in the context of a popular feast (Fig. 4). In other cases, such as the elaboration of matrasses with *Zea mays* L. inflorescence bracts or that of sheets with *Cannabis sativa* L. fibres, the uses have been completely abandoned.

Even in some trades that are declining, a certain degree of vitality persists: the elaboration of musical instruments for the orchestra (named *cobla*) that plays typical Catalan dances (called *sardana*) and for other kind of traditional musical events, importantly involving *Ziziphus jujuba* Mill. (Fig. 4) continues to be alive, even if their artisans must have another profession to make a living.

Apart from these declining uses, some other persist almost to the same extent as always. These are uses linked to everyday life mostly in rural areas, such as those related to agricultural practices (in homegardens or elsewhere), e.g., tutoring some cultivated plants (Fig. 3). Another kind of folk plant knowledge to persist is that related to ludic aspects, including oral literature. People continue to play with plants and to use sayings and proverbs involving the vegetal world (even to inventing new ones). Additionally, some plant uses directly linked to commercial issues have also a high degree of persistence, such as those related to basketry (Fig. 1) or Christmas/New Year decoration (Fig. 4).

Finally, some popular traditions linked to plants are still in use, but have changed in some senses. As an example, some plants are hung in or near the doors of many rural houses (especially in mountain areas), as was commonly done since time immemorial, such as *Carlina acanthifolia* (Fig. 4). Nevertheless, no one believes nowadays (even if many people did a few decades ago) that these plants are protective for people and animals in the house. In this case, these plants now play a decorative role, with the associated interest that people know that this current use derives from a quite different ancient one. The basket elaboration mentioned in the last paragraph and similar activities, such as some related to textile, dyeing and handicraft issues, have also experienced, at least to some extent, this change. In past times, baskets, carpets, blankets, forks, spoons and other objects were elaborated with plant material simply for their use within the house, but now most such pieces produced are addressed to decorative and touristic purposes, comporting some commercial revenues.

## Conclusions

The important number of species claimed to be used (552, with 4137 use reports) by the 769 informants interviewed in the territories studied for the purposes comprised in this paper show that popular knowledge on plants goes far beyond food and medicinal applications, which had been the most traditionally studied in ethnobotanical surveys, probably because they are the most apparent and the most preserved plant utilisations. This means that a large and diverse panoply of traditional uses, which are apparently secondary in our industrialised societies, such as examples as different as basket elaboration and oral literature, remains active and, when this is not so, appreciated and remembered by people.

To summarize, we believe that the data in this study show that a robust set of knowledge and practices in the field of non-food and non-medicinal plant uses persists in the European industrialised area studied, indicating that these utilisations of plants, which to date have been the object of scarce attention in ethnobotanical research, are relevant -we would say fully necessary- for a complete life, even in a modern society and even if they are not directly related to health issues. Additionally, and not forcibly negatively, although this means a reorientation in the tradition, some changes in popular uses (mostly those related to handicrafts) currently imply a certain complementary income for some people and may represent a potential for future commercial activities with economic significance in rural communities.

Further ethnobotanical studies in this currently still rather neglected field are encouraged in order to compile an important part of natural and cultural heritage not sufficiently considered up to now, and to assess what actions are needed to preserve this knowledge, particularly in cases of dangerously declining real current practice.

## Abbreviations

AE, Alt Empordà district; BC, Herbarium of Botanical Institute of Barcelona; BCN, Herbarium of the Centre de Documentació de Biodiversitat Vegetal, Universitat de Barcelona; BI, Balearic Islands; CA, Catalonia; F_IC_, Informant consensus factor; FO, Formentera island; MA, Mallorca island; MO, Montseny massif; RI, Ripollès district; UR, Use reports
